# A Quantitative Systems Pharmacology Platform Reveals NAFLD Pathophysiological States and Targeting Strategies

**DOI:** 10.3390/metabo12060528

**Published:** 2022-06-07

**Authors:** Daniel E. Lefever, Mark T. Miedel, Fen Pei, Johanna K. DiStefano, Richard Debiasio, Tong Ying Shun, Manush Saydmohammed, Maria Chikina, Lawrence A. Vernetti, Alejandro Soto-Gutierrez, Satdarshan P. Monga, Ramon Bataller, Jaideep Behari, Vijay K. Yechoor, Ivet Bahar, Albert Gough, Andrew M. Stern, D. Lansing Taylor

**Affiliations:** 1 Drug Discovery Institute, University of Pittsburgh, Pittsburgh, PA 15261, USA; del53@pitt.edu (D.E.L.); mmiedel@pitt.edu (M.T.M.); rid14@pitt.edu (R.D.); tos8@pitt.edu (T.Y.S.); mas386@pitt.edu (M.S.); vernetti@pitt.edu (L.A.V.); als208@pitt.edu (A.S.-G.); smonga@pitt.edu (S.P.M.); yechoorv@pitt.edu (V.K.Y.); bahar@pitt.edu (I.B.); gough@pitt.edu (A.G.); 2 Department of Computational and Systems Biology, School of Medicine, University of Pittsburgh, Pittsburgh, PA 15260, USA; fep7@pitt.edu (F.P.); mchikina@pitt.edu (M.C.); 3 Diabetes and Fibrotic Disease Unit, Translational Genomics Research Institute TGen, Phoenix, AZ 85004, USA; jdistefano@tgen.org; 4 Pittsburgh Liver Research Center, University of Pittsburgh, Pittsburgh, PA 15261, USA; 5 Department of Pathology, School of Medicine, University of Pittsburgh, Pittsburgh, PA 15261, USA; 6 McGowan Institute for Regenerative Medicine, University of Pittsburgh, Pittsburgh, PA 15203, USA; 7 Division of Gastroenterology Hepatology and Nutrition, Department of Medicine, School of Medicine, University of Pittsburgh, Pittsburgh, PA 15261, USA; bataller@pitt.edu (R.B.); jab31@pitt.edu (J.B.); 8 UPMC Liver Clinic, University of Pittsburgh Medical Center, Pittsburgh, PA 15213, USA; 9 Division of Endocrinology and Metabolism, Department of Medicine, School of Medicine, University of Pittsburgh, Pittsburgh, PA 15203, USA

**Keywords:** liver, non-alcoholic fatty liver disease, NAFLD, metabolic-associated fatty liver disease, MAFLD, microphysiology systems, MPS, drug discovery, quantitative systems pharmacology, QSP, connectivity map, CMap, drug repurposing, network proximity, non-alcoholic steatohepatitis, NASH, fibrosis, lobular inflammation, steatosis, targeting disease states, drug combinations

## Abstract

Non-alcoholic fatty liver disease (NAFLD) has a high global prevalence with a heterogeneous and complex pathophysiology that presents barriers to traditional targeted therapeutic approaches. We describe an integrated quantitative systems pharmacology (QSP) platform that comprehensively and unbiasedly defines disease states, in contrast to just individual genes or pathways, that promote NAFLD progression. The QSP platform can be used to predict drugs that normalize these disease states and experimentally test predictions in a human liver acinus microphysiology system (LAMPS) that recapitulates key aspects of NAFLD. Analysis of a 182 patient-derived hepatic RNA-sequencing dataset generated 12 gene signatures mirroring these states. Screening against the LINCS L1000 database led to the identification of drugs predicted to revert these signatures and corresponding disease states. A proof-of-concept study in LAMPS demonstrated mitigation of steatosis, inflammation, and fibrosis, especially with drug combinations. Mechanistically, several structurally diverse drugs were predicted to interact with a subnetwork of nuclear receptors, including pregnane X receptor (PXR; NR1I2), that has evolved to respond to both xenobiotic and endogenous ligands and is intrinsic to NAFLD-associated transcription dysregulation. In conjunction with iPSC-derived cells, this platform has the potential for developing personalized NAFLD therapeutic strategies, informing disease mechanisms, and defining optimal cohorts of patients for clinical trials.

## 1. Introduction

Nonalcoholic fatty liver disease (NAFLD), also known as metabolic dysfunction associated fatty liver disease (MAFLD) [1], is a heterogeneous disease with a complex pathogenesis involving several diverse signaling cues from the environment, the microbiome, metabolism, comorbidities such as type 2 diabetes, and genetic risk factors [2,3]. NAFLD comprises a spectrum of progressive disease states from simple hepatic steatosis (fatty liver) termed NAFL to a more serious condition, nonalcoholic steatohepatitis (NASH), involving inflammation, hepatocyte damage (i.e., ballooning), and often pericellular fibrosis [4,5]. NASH itself is a risk factor for cirrhosis and end-stage liver disease requiring liver transplantation [6] and for hepatocellular carcinoma (HCC) that can progress insidiously before cirrhosis is diagnosed [7]. The prevalence of NAFLD is approximately 25% across adult populations worldwide, with the proportion of those with NASH predicted to increase over the next decade [6].

Despite the major public health problem NAFLD presents and the economic burden it exacts [8], no drug treatments have been approved [5,9]. Although significant progress towards an understanding of NAFLD molecular pathogenesis has been made, our current knowledge base is insufficient to accurately predict disease progression and response to emerging therapies, even in similar patient cohorts. To address this unmet need, the research community has adopted systems-based approaches [10,11,12,13,14], such as quantitative systems pharmacology (QSP) [15]. QSP comprehensively and unbiasedly integrates molecular, cell, and clinical data to generate predictive models of disease progression. These computational models are then tested and iteratively refined using experimental models to identify specific signaling networks and predictive biomarkers mechanistically linked to pathogenesis (i.e., NAFLD) [16]. Intrinsic to QSP is the implementation of human microphysiological systems (MPS) that recapitulate key aspects of NAFLD pathogenesis and, in conjunction with iPSC technology [17], can ultimately be used to address those issues confounded by patient heterogeneity, thus serving as an important complement to animal models. An overarching goal of implementing a QSP approach is to identify NAFLD subtypes having distinguishable mechanisms of disease progression [2]. It is hypothesized that a molecular-based disease sub-classification that has remained elusive thus far will enable precision medicine and therapeutic advances for targeting patient cohorts with specific drug combinations [2].

**Figure 1:**
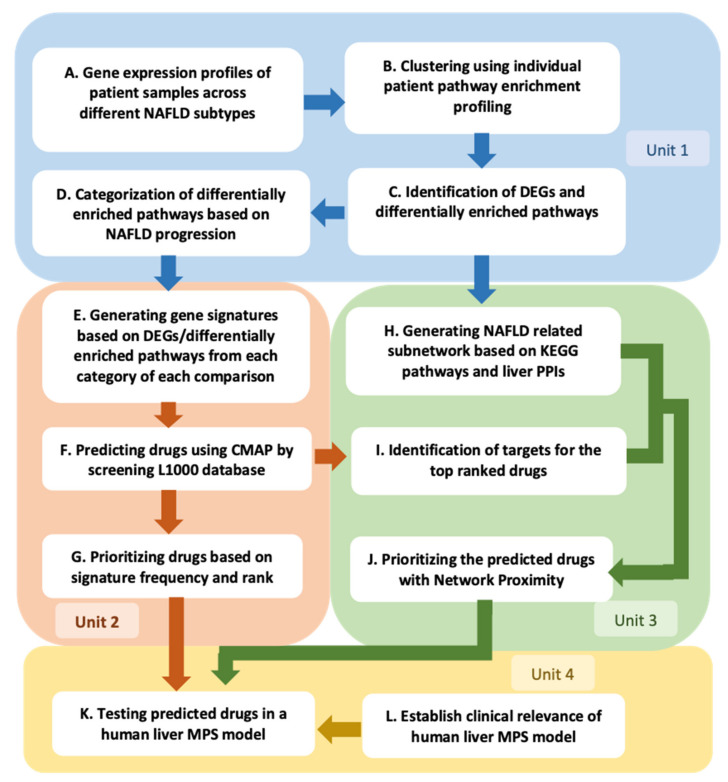
Workflow associating NAFLD subtypes with gene expression signatures to computationally predict and prioritize drugs for testing in a patient-derived microphysiological model of disease progression. Four integrated units are shown, each comprised of a set of steps detailed in the Methods and Results. Unit 1A–D identifies and clusters individual patient hepatic gene expression and enriched pathway profiles associated with clinical subtypes and categorizes the differentially enriched pathways among these clusters (Figure 2 and Figure 3; Tables S2 and S3, and Data files S1 and S2) within our current framework of NAFLD pathophysiology [2,5]. The rationale is presented in the Results for using clusters based on individual patient pathway enrichment profiles as an alternative to the clinical classifications (compare Figure 3, Figures S1 and S2) for determining both differentially expressed genes and enriched pathways between different stages of disease progression. Unit 2E–G generates disease progression-based gene expression signatures (Table S4; Data file S3) and, using the Connectivity Map (CMap) databases, identifies drugs that can normalize these signatures (Table 1 and Table S5; and Data files S4 and S5). The highly integrative Unit 3 H-J maps known protein targets of the predicted drugs from Unit 2 to an NAFLD subnetwork encompassing protein targets from the gene expression analysis within Unit 1 (Figure S6; Table S7, and Data file S6). A network proximity score is then calculated that helps prioritize candidate drugs identified by CMap analysis for experimental testing based on the proximity of their targets to the NAFLD subnetwork (Table S8; Data file S7). In Unit 4K, the effects of the prioritized drugs on a diverse set NAFLD–associated biomarkers in a human MPS, independently shown to recapitulate critical aspects of NAFLD progression (Unit 4L) (Figure 4, Figures S4 and S5), are determined (Figure 5 and Figure 6). Table S1 provides an index of tables, figures, and data files associated with each step.

**Figure 2:**
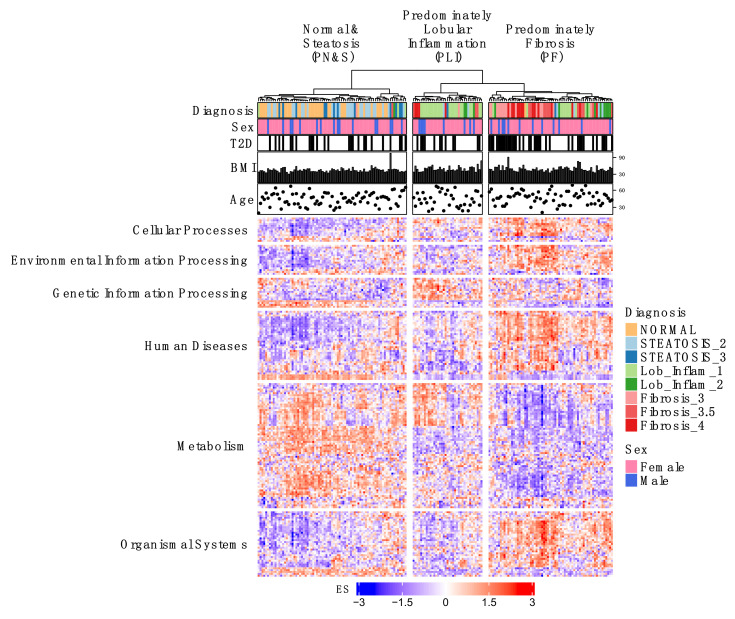
Individual patient liver transcriptome analysis yields distinct clusters based on their Kyoto Encyclopedia of Genes and Genomes (KEGG) pathway enrichment profiles. The heatmap shows the hierarchical clustering of the liver KEGG pathway enrichment profiles (*columns*) from individual patients, determined by RNA sequencing and gene set variation analysis (GSVA) using MSigDB v7.0 C2 KEGG pathways [24] (see Methods). Pathways (*rows*) are grouped according to the top-level KEGG hierarchical classifications (labeled along the left ordinate) to which they belong. The color represents the enrichment score (ES; see the *color-coded bar* under the heatmap), which reflects the degree to which a pathway is over- or under-represented within that individual patient sample (see [25]). The plots above the heatmap show the patient metadata: the top two bars indicate the color-coded diagnosis (see panel on the *right*) and patient sex, the third indicates if the patient has been diagnosed with type 2 diabetes (T2D) (*black bars*), and the additional two plots show the body mass index (BMI) and age of the patient. The clinical subtype distribution for each of the three clusters (PN&S, PLI, PF) is shown in Table S2. More details on this analysis, including the specific pathway information and patient metadata, can be found in the associated R notebooks [26].

Herein, we describe the implementation of a QSP-based platform [15,18] (Figure 1, Table S1) that starts with the computational analysis of individual patient-derived hepatic RNA-seq data encompassing a full spectrum of NAFLD disease states from simple steatosis, to NASH, to advanced fibrosis and cirrhosis, and including associated comorbidities such as type 2 diabetes (T2D) [19]. This analysis has enabled us to associate distinct clusters of individual patient gene expression and pathway enrichment profiles with three NAFLD sub-classifications: predominantly normal and steatosis (PN&S); predominantly lobular inflammation (PLI), typical of NASH early stages; and predominantly fibrosis (PF), reflecting NASH progression (Figure 2). Approved or investigational drugs predicted to potentially revert the resulting disease gene signatures were identified from the Library of Integrated Network-Based Cellular Signatures (LINCS) L1000 database [20,21,22] and prioritized for experimental testing using two complementary approaches (Figure 1 Units 2 and 3). One approach is based on the frequency of appearance and rank of the candidate drug across multiple signatures (Figure 1 Unit 2). The other prioritization approach considers an NAFLD subnetwork independently constructed from genes differentially expressed during NAFLD progression and uses network proximity [23] to rank drugs according to the proximity of their targets to this subnetwork (Figure 1 Unit 3). The prioritized candidate drugs are evaluated in a clinically relevant human biomimetic liver MPS model of NAFLD progression employing a diverse panel of biomarkers (Figure 1 Unit 4; Figure S1) to: (1) provide experimental proof-of-concept for the computation-based predictions; (2) identify drugs and combinations that could form the basis for developing new N非29AFLD therapeutic strategies; and (3) gain mechanistic insights into the heterogeneity of NAFLD pathophysiology, which will enable precision medicine.

## 2. Results

### 2.1. Individual Patient KEGG Pathway Enrichment Profiles Cluster According to Predominant NAFLD Subtypes

To help distill NAFLD complexity at the molecular level and associate hepatic signaling network dysregulation with clinical subtypes, we performed an unsupervised gene set variation analysis (GSVA) derived from 182 individual patient liver biopsies representing different stages of NAFLD [19] that included 36 normal, 46 steatosis, 50 lobular inflammation, and 50 fibrosis (Figure 1A,B). The resulting KEGG pathway enrichment profiles were then subjected to hierarchical clustering with the dendrogram cut at the third level to create three distinct clusters (see Methods) that were each enriched in different stages of disease (Figure 1B and Figure 2; Table S2; Methods). The first cluster is composed of 44% normal patients and 48% patients with simple steatosis (NAFL), termed predominantly normal and steatosis (PN&S), highlighting the challenge of distinguishing these two cohorts by gene expression analysis alone when inflammation is not discernable; the second cluster is predominated by patients with lobular inflammation (70%) with little or no fibrosis, termed predominantly lobular inflammation (PLI); and the third is predominantly comprised of patients with advanced disease having fibrosis (61%), termed predominantly fibrosis (PF) (Figure 2; Table S2). The sample clustering is significantly associated (Pearson’s Chi-squared Test) with the NAFLD subclass (*p* < 2.2 × 10^−16^) and T2D status (*p* = 0.01). Figure 2 also shows that the distributions of sex, body mass index (BMI), and age are similar across the different clusters. In contrast, the occurrence of T2D in cluster PF (55%) is higher than in clusters PN&S (32%) and PLI (32%), corroborating that among individuals with T2D and NAFLD, the prevalence of NASH and advanced fibrosis is enriched when compared to nondiabetics with NAFLD, as observed in independent analyses of this particular cohort [19] and other cohorts [27,28,29]. This is most evident among the 40 patients diagnosed with fibrosis within the PF cluster, with 78% having T2D (Table S2). 

We next investigated in more detail the association between distinct pathway enrichment profiles (i.e., molecular disease phenotypes) and clinical subtypes by determining the differential pathway enrichment profiles of the pairwise comparisons among the three clusters and among the corresponding clinical subtypes (Figure 1C). 

**Figure 3:**
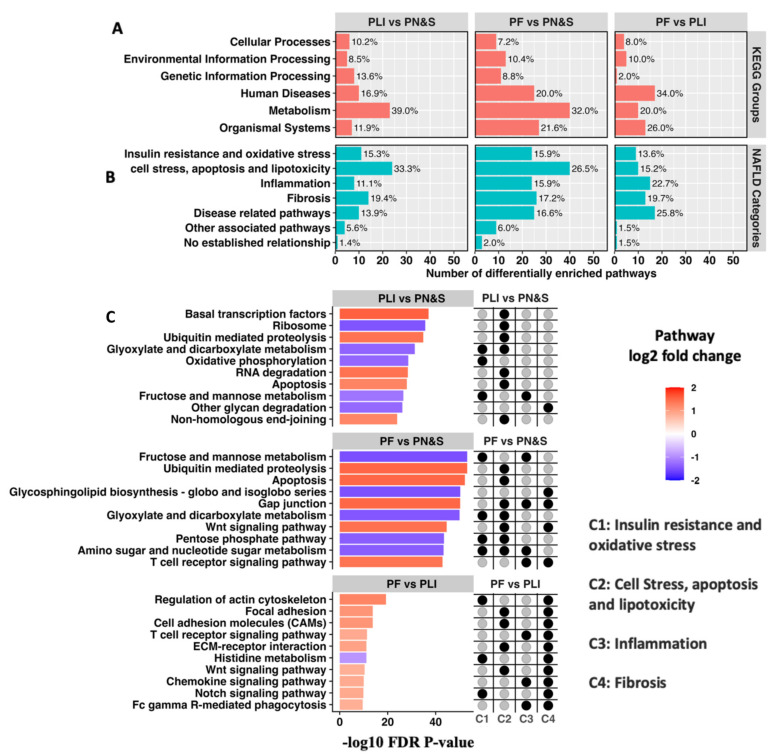
Distribution of differentially enriched pathways and their respective KEGG groups and NAFLD categories among the pairwise cluster comparisons defined in Figure 2. The number of differentially enriched pathways identified between the PLI vs. N&S, PF vs. N&S, and PF vs. PLI pairwise comparisons were 59, 125, and 50, respectively (adj. *p*-value < 0.001). Their distribution (and percent contribution) with respect to KEGG groups (**A**) and NAFLD categories (**B**) are detailed in Table S3 and Data file S1. The top ten differentially enriched pathways for each comparison (ranked by the FDR adjusted *p*-values through the linear modeling equivalent of a two-sample, moderated *t*-test) are shown along with their association (black circles) with NAFLD categories C1–4 (as indicated and defined in the Main Text) (**C**). The colors of the bars represent the directionality and relative enrichment of each pathway for each of the pairwise comparisons.

The pairwise cluster comparisons of PLI vs. PN&S, PF vs. PN&S, and PF vs. PLI gene and pathway expression data yielded a total of 139 unique differentially enriched pathways (FDR *p*-value < 0.001) (Figure 1C; Table S3; Data file S1). Analogously, the pairwise clinical subtype comparisons of lobular inflammation vs. normal and steatosis (Lob vs. N&S), fibrosis vs. normal and steatosis (Fib vs. N&S), and fibrosis vs. lobular inflammation (Fib vs. Lob) gene and pathway expression data yielded a total of 140 unique differentially enriched pathways (FDR *p*-value < 0.001) (Table S3; Data file S1). The distributions of these differentially enriched pathways within their respective top-level KEGG hierarchical classifications in each pairwise comparison are presented in Figure 3A and Figure S1A, respectively. Overall, these distributions are consistent with the intrinsic heterogeneity of NAFLD that reflects the diverse but convergent impacts of the environment, metabolism, comorbidities, and genetic risk factors [2]. More specifically, many of these differentially enriched pathways can be associated with at least one of four categories that comprise our current conceptual framework of NAFLD progression (Figure 1D, Methods): (C1) insulin resistance and oxidative stress; (C2) cell stress, apoptosis, and lipotoxicity; (C3) inflammation; and (C4) fibrosis (Figure 3B and Figure S1B) [2,5]. Apart from these four main categories, other pathways have been observed that are less directly associated with NAFLD or metabolic syndrome (Figure 1D, Figure 3B and Figure S1B). 

The 10 most differentially enriched pathways for all patient subgroup pairwise comparisons, and their association with the disease processes within these four categories (C1–C4), are shown in Figure 3C and Figure S1C. The 10 pathways for the PF vs. PN&S and the PLI vs. PN&S cluster-based comparisons, and the Fib vs. N&S and the Lob vs. N&S clinical subtype comparisons are consistent with the metabolic underpinning, and the resultant cellular stress and inflammatory response intrinsic to NAFLD pathogenesis. Complementarily, the differentially enriched pathways within the comparisons between PF vs. PLI and between Fib vs. Lob are consistent with fibrosis being the widely recognized hallmark of disease progression in NASH (Figure 3C and Figure S1C). The majority of the top 10 differentially enriched pathways in these comparisons have been shown to have a role in hepatic fibrosis [30,31,32,33,34,35,36,37] with several involved in hepatic stellate cell activation [30,31,32]. The majority of differentially enriched pathways derived from the unsupervised clusters are concordant with those derived from the clinical subtypes per se (Figure S2), corroborating an association of these pathways with NAFLD progression. A meta-analysis extending the unsupervised cluster comparisons to three independent NAFLD patient cohorts further supports an association of many of these differentially enriched pathways with NAFLD progression (Figure S3). The fraction of the top 10 differentially enriched pathways playing a role in multiple disease categories in the PF vs. PLI comparison was greater than the fractions in the other two comparisons, indicative of enhanced disease complexity during progression (Figure 3C). Details of the full list of differentially enriched pathways for each comparison can be found in Table S3 and Data file S1. Together, the analysis of this transcriptomic dataset appears to have corroborated the clinical relevance of these differentially enriched pathways in the context of the current conceptual framework of NAFLD progression [2,5].

Although each of these identified differentially enriched pathways has the potential to be a drug target, their large number and diversity, the prospect of redundancy, and the uncertainty regarding their individual contribution to NAFLD pathogenesis, especially across a heterogeneous patient population, all present challenges to translating this information into revealing pathophysiological mechanisms and informing therapeutic strategies. To help conceptualize this translational objective, we suggest that differentially expressed gene (DEG) signatures that map to differentially enriched pathways involved in the disease processes comprising the four NAFLD categories C1–C4 (see below and Figure 1E; Table S4; Data file S3; Methods) mirror emergent disease-specific networks (i.e., disease states) at different stages of disease progression. We hypothesize that pharmacologically normalizing these gene signatures using the integrative approach outlined in Figure 1E–L and below will modify disease progression in a clinically relevant human MPS model of NAFLD.

### 2.2. Initial Prediction and Testing of Drugs in a Human Liver MPS Model of NAFLD

To predict drugs/small molecules that modulate individual components of NAFLD progression, we initially focused on the DEGs (Data file S2) that mapped to the categorized (four NAFLD categories, C1–C4; Methods) differentially enriched pathways (Figure 1D–E; Table S3; Data file S1; Methods) identified above in each of the three comparisons of unsupervised clusters (i.e., PLI vs. PN&S, PF vs. PN&S, and PF vs. PLI) resulting in a total of 12 gene signatures (Table S4; Data file S3; Methods). Each of these 12 gene signatures was then used as input to perform connectivity mapping (CMap) on the LINCS database (see [20] and Methods). 

CMap connects the DEG signature between different disease states (including the non-disease state) to drugs and other pharmacologically active compounds predicted to normalize the disease-associated gene signature (see Methods) [20,21,22]. In the context of this study, the output of CMap [20,21,22] enables the pharmacologic testing of the hypothesis that normalization of the gene signatures between two disease states will halt or perhaps reverse disease progression in an experimental human NAFLD model (see below; Methods). Since a key objective is to identify drugs that can be repurposed for preventing NAFLD progression, we focused on CMap outputs present in DrugBank (see Methods) that could promote the reversion of the disease-associated gene signature in each NAFLD category (Methods; Figure 1F). For our initial study using the 2017 LINCS database [20], we selected the top 20 drugs (ranked by their most negative CMap score among all instances for that particular drug, see Methods) for each of the 12 queries, resulting in 106 unique predicted drugs, 35 of which appeared as an output in more than one query (Figure 1G; Table S5; Data file S4). Given the complex interplay among dysregulated metabolic pathways, oxidative and ER stress, inflammation, and fibrosis during NAFLD progression, our initial prioritization of 25 drugs focused on those predicted to modulate multiple gene expression signatures (Figure 1G; Table S5). Enriched in this set are drugs with targets known to be associated with NAFLD and with the potential to act pleiotropically to modulate several pathways. For example, vorinostat is predicted to normalize 5 of the 12 signatures focused primarily on inflammation and fibrosis, and previous studies in rodent models of NAFLD suggested efficacy with other HDAC inhibitors [38,39].

**Figure 4:**
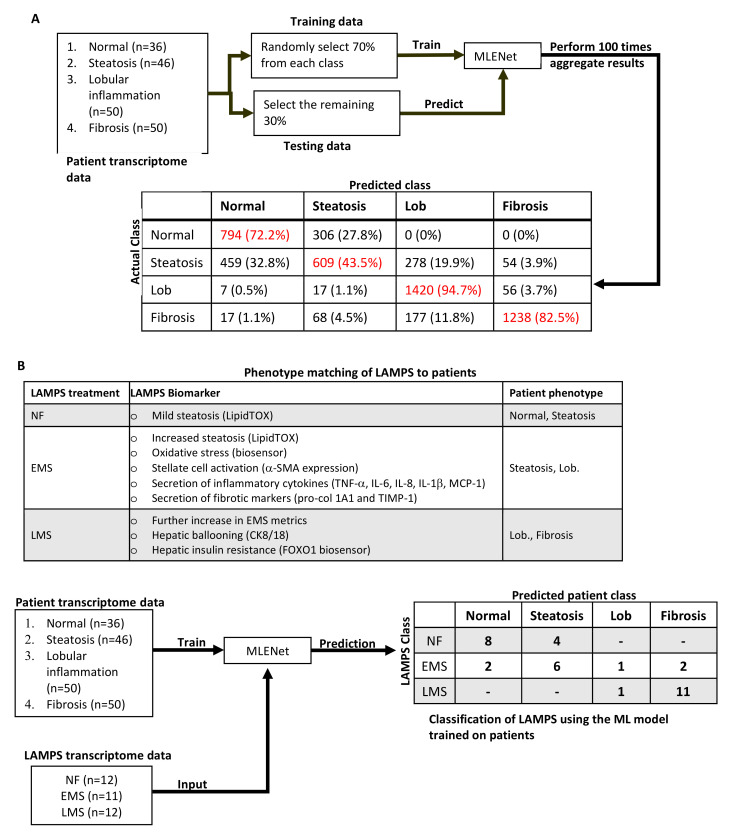
Unbiased machine learning model of patient transcriptomic data identifies and predicts congruent clinical phenotypes within LAMPS. (**A**) The bootstrapping procedure used to develop and validate the transcriptome-based machine learning model (MLENet) capable of differentiating and predicting 4 NAFLD patient classifications (see Methods) (red indicates the clinically defined true positives). The average sensitivity across the bootstrapping instances (numbers in parenthesis are standard deviations) are: 0.66 (0.11), 0.64 (0.12), 0.77 (0.08), 0.93 (0.07); average specificity 0.93 (0.03), 0.83 (0.03), 0.98 (0.02), 0.95 (0.03) for normal, steatosis, Lob, and fibrosis, respectively. (**B**) The workflow and table of outcomes from implementing MLENet to identify and predict congruent NAFLD patient phenotypes from LAMPS transcriptomic analytes generated under normal fasting (NF); early metabolic syndrome (EMS); or late metabolic syndrome (LMS) conditions (see Methods). The phenotype matching of LAMPS to patients results from extensive parallel biochemical and imaging analyses [41], indicating that the three different media conditions drive distinct phenotypes congruent with clinical phenotypes of NAFLD progression and are independently consistent with the machine learning approach.

We next used our LAMPS model of NAFLD to test the predicted drugs. The LAMPS model comprises an all-human cell platform containing primary hepatocytes and liver sinusoidal endothelial cells (LSECs) as well as Kupffer (differentiated THP-1) and stellate (LX-2) cell lines layered in a microfluidic device that recapitulates several key structural features and functions of the human liver acinus [16,40,41] (Figure 1K and Figure S4A; Methods). The LAMPS model has been tested and reproduced by the Texas A&M Tissue Chip Validation Center (Tex-Val), one of the National Center for Advancing Translational Sciences (NCATS) funded Tissue Chip Testing Centers (TCTC) [42]. We have recently demonstrated that this model system recapitulates critical aspects of NAFLD progression, including lipid accumulation, stellate cell activation, and the production of pro-inflammatory cytokines and fibrotic markers, using media containing key NAFLD drivers, including increased levels of glucose, insulin, and free fatty acids [16,41] (Figure S4B; Methods). To gain further evidence supporting the clinical relevance of the LAMPS NAFLD model, we implemented a machine learning approach based on transcriptomic analysis of the 182 patient cohort [19] described in Figure 2 and Table S2 (Methods; Figure 1L). We first trained a multinomial logistic regression with an elastic net penalization model (MLENet) using nested cross-validation to successfully differentiate among four clinical classifications of NAFLD (Figure 4A). The final model used 71 genes, with 80% of these having prior association with NAFLD (Data file S10). Using this patient-based model, we then classified the transcriptome of individual LAMPS under three media conditions, normal fasting (NF), early metabolic syndrome (EMS), and late metabolic syndrome (LMS), as shown in Figure 1L and Figure 4B and the Methods. At the transcriptome level, progression of NAFLD in LAMPS upon media treatment mimics disease progression observed in patients, independently corroborating the biomarker and imaging data (Figure 1L and Figure 4B). 

**Figure 5:**
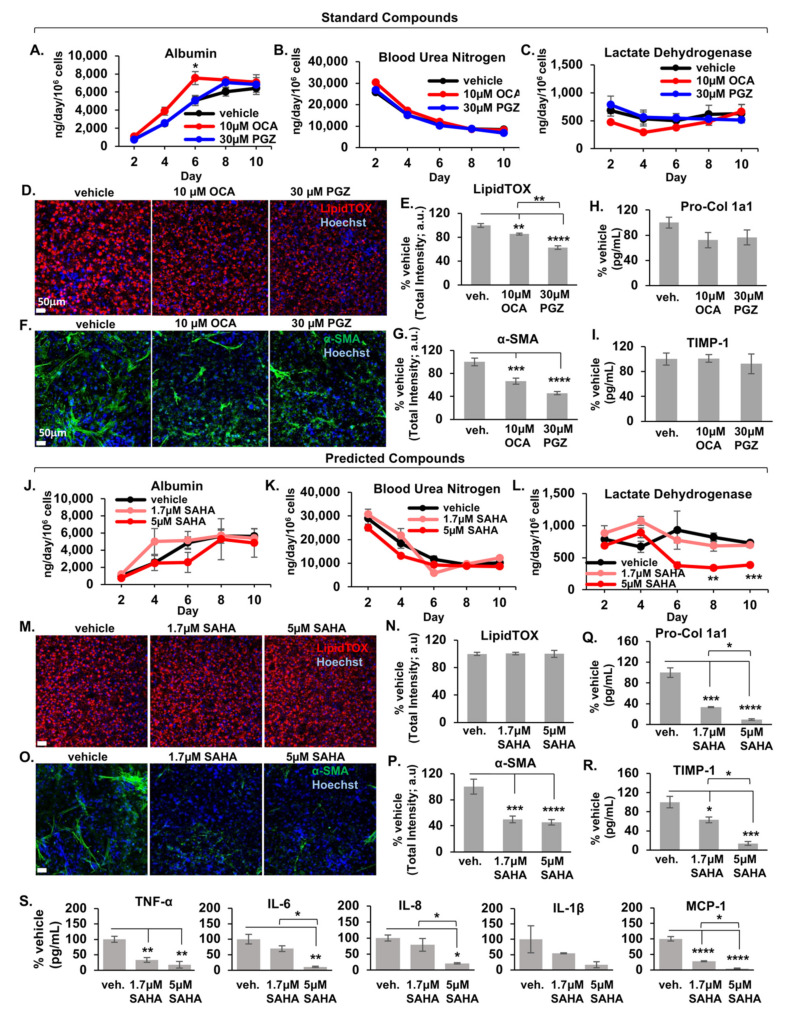
Control and predicted drugs reduce different NAFLD disease phenotypes in LAMPS models treated with EMS media. LAMPS models were maintained for 10 days in Early Metabolic Syndrome (EMS) media containing either vehicle control, 10 µM obeticholic acid (OCA) and 30 µM Pioglitazone (PGZ) [standard compounds], or vorinostat (suberoylanilide hydroxamic acid; SAHA) at 1.7 µM or 5 µM [predicted compounds]. A panel of metrics was examined to monitor disease-specific phenotypes. For standard drugs, albumin, blood urea nitrogen, and lactate dehydrogenase curves throughout the time course show similar profiles throughout the time course between vehicle and drug treatment groups, suggesting no overt model cytotoxicity or loss of function (**A**–**C**). At the day 6 timepoint, there was a significant increase in albumin secretion in the OCA group; however, no further significant increases in albumin output were observed at later time points (days 8 and 10). At day 10, there was a significant decrease in steatosis (**D**,**E**; LipidTOX^TM^ intensity) and stellate cell activation (**F**,**G**; α-SMA intensity) for both OCA and PGZ groups compared to vehicle. Panels (**D**,**F**) display representative 20X image Day 10 LipidTOX^TM^ (**D**) and α-SMA (**F**) images of LAMPS. Scale bar; 50 μm. There was no significant change in the secreted levels of the pro-fibrotic markers Pro-Col 1a1 (**H**) TIMP-1 (**I**) in either treatment group compared to vehicle. For the predicted drug vorinostat (SAHA), albumin and blood urea nitrogen curves show no significant differences between vehicle and treatment groups (**J**,**K**), suggesting that these drug treatments do not result in loss of model functionality; however, a significant decrease in LDH secretion (**L**) at days 8 and 10 in the 5 µM vorinostat treatment group, suggesting decreased cytotoxicity. This was further supported by the significant decrease in stellate cell activation (**O**,**P**; α-SMA intensity), production of the pro-fibrotic markers pro-collagen 1a1 and TIMP-1 (**Q**,**R**), and inflammatory cytokine production (**S**) observed in the vorinostat group. In contrast, vorinostat does not reduce lipid accumulation compared to vehicle control (**M**,**N**), indicating no effect on steatosis. Panels (**M**,**O**) display representative 20X image Day 10 LipidTOX^TM^ (**D**) and α-SMA images of LAMPS under each treatment condition. Scale bar; 50 μm. For each control and drug treatment group, n = 3 chips were analyzed and plotted ± SEM for each assay and statistical significance was assessed using a One-Way ANOVA with Tukey’s test (* *p* < 0.05; ** *p* < 0.01; *** *p* < 0.001; **** *p* < 0.0001).

We then examined the effects of two control drugs that have shown appreciable clinical benefits in NAFLD clinical trials, obeticholic acid (OCA) [43,44] and pioglitazone (PGZ) [45], using the LAMPS experimental model (Figure 1K and Figure 5). LAMPS were maintained for 10 days in EMS media containing either the indicated concentration of drug or DMSO vehicle control. EMS conditions were selected since biomarker and imaging analysis indicate that steatosis, inflammation, and fibrosis are progressively induced during the 10-day testing period [41]. We determined drug concentrations to test in LAMPS guided by the concentrations indicated in the LINCS L1000 database, reported PK/PD, and the absence of cytotoxicity at these concentrations during pre-testing in primary hepatocytes (Table S6). In addition, we determined the amount of each compound that was adsorbed by the PDMS component of the LAMPS device (Table S6). We examined a panel of metrics to monitor disease-specific phenotypes, including model functionality (albumin and blood urea nitrogen production), cytotoxicity (lactate dehydrogenase secretion), hepatocellular steatosis (LipidTOX^®^ labeling), stellate cell activation (α-smooth muscle actin staining), and the production of a panel of pro-inflammatory cytokines (TNF-α, IL-6, IL-8, IL-1α, and MCP-1) and fibrotic markers (Pro-collagen 1A1 and TIMP-1) [41] (Figure S4B).

LAMPS models were maintained for 10 days in EMS media containing 10 µM OCA, 30 µM PGZ, or vehicle control (Figure 5). Throughout the experimental time course, albumin, blood urea nitrogen, and lactate dehydrogenase showed similar secretion profiles between vehicle control and drug treatment groups (Figure 5A–C), suggesting no hepatocellular damage or loss of function. At the day 6 timepoint (Figure 5A), there was a significant increase in albumin secretion in the OCA group; however, no further significant increases in albumin output were observed at later time points (days 8 and 10). However, there was a significant decrease in LipidTOX^®^ and α-SMA staining intensity in the OCA and PGZ treatment groups compared to vehicle control, demonstrating that both hepatocellular steatosis (Figure 5D,E) and stellate cell activation (Figure 5F,G) were reduced. Although there was a ~20% decrease in secretion of the pro-fibrotic marker Pro-collagen 1a1 (Figure 5H) with treatment of OCA or PGZ, this decrease was not statistically significant, similar to other previous studies examining collagen 1 gene expression and secretion in response to treatment with OCA and PGZ [46,47]. In addition, there was also no significant change in the secreted levels of TIMP-1, another pro-fibrotic marker, in any of the treatment groups compared to vehicle (Figure 5I).

We next examined the effect of the histone deacetylase (HDAC) inhibitor, vorinostat (abbreviated SAHA), the highest ranking drug predicted from our initial CMap analysis (Figure 1K and Figure 5J–S; Table S5). LAMPS models maintained for 10 days in EMS disease media contained either vorinostat (1.7 µM or 5 µM) or DMSO vehicle control. As shown in Figure 5, albumin and blood urea nitrogen curves showed no significant differences between vehicle and drug treatment groups (Figure 5J,K), suggesting that these drug treatments do not induce appreciable loss of hepatic functionality. There was a significant decrease in LDH secretion (Figure 5L) at days 8 and 10 in the 5 µM vorinostat treatment group, suggesting that treatment with this drug alleviates disease media-induced cytotoxicity. This result is further supported by the overall significant decrease in the day 10 measurements of stellate cell activation (Figure 5O,P; α-SMA intensity), production of the pro-fibrotic markers pro-collagen 1a1 and TIMP-1 (Figure 5Q,R), and inflammatory cytokine production (Figure 5S) observed in the vorinostat treatment group. In contrast to PGZ and OCA, and despite its significant effect on profibrotic markers, vorinostat treatment did not appreciably alleviate lipid accumulation at day 10 (Figure 5M,N), indicating no significant effect on steatosis.

**Figure 6:**
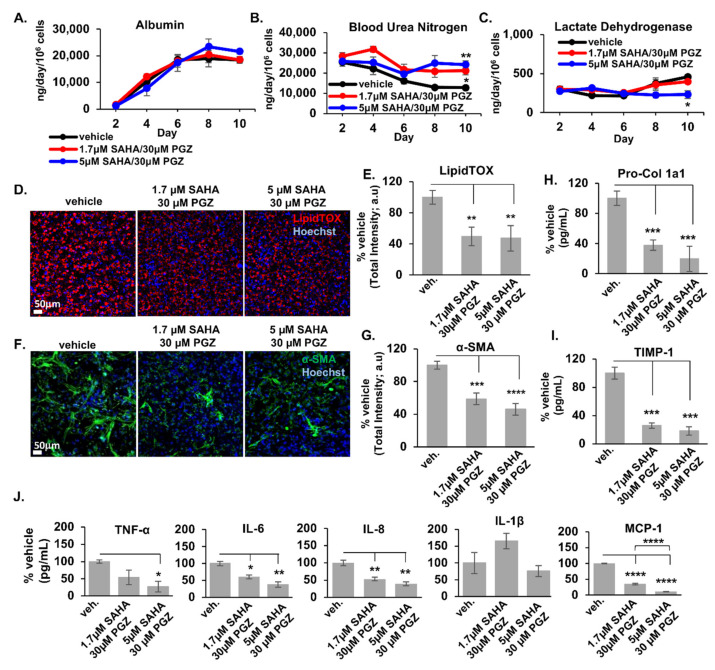
Pioglitazone and vorinostat used in combination result in the reduction of steatosis and stellate cell activation as well as the secretion of pro-fibrotic markers and production of inflammatory cytokines in LAMPS models treated with EMS media. LAMPS models were maintained for 10 days in NAFLD disease media containing combinations of pioglitazone (30 µM) and vorinostat (1.7 µM or 5 µM) or DMSO vehicle control. A panel of metrics was examined to monitor disease-specific phenotypes under these treatment conditions. While albumin secretion profiles show no significant differences between vehicle and drug treatment groups, suggesting that these drug combinations do not result in loss of model functionality (**A**), a significant increase in urea nitrogen secretion is observed in both drug combination groups compared to control, suggesting increased model metabolic activity (**B**). In addition, like the LDH profile in Figure 5, there is a significant decrease in LDH secretion (**C**) in the 5 µM vorinostat treatment group, suggesting a reduction in cytotoxicity. Compared to the contrasting effects observed in the individual drug testing studies shown in Figure 5, we observe an overall decrease in both lipid accumulation (**D**,**E**) and stellate cell activation (**F**,**G**), as well as in the production of the pro-fibrotic markers pro-collagen 1a1 and TIMP-1 (**H**,**I**) and inflammatory cytokine production (**J**) when pioglitazone and vorinostat are used in combination. Panels (**D**,**F**) display representative 20X image Day 10 LipidTOX^TM^ (**D**) and α-SMA (**F**) images of LAMPS under each treatment condition. Scale bar; 50 μm. For each control and drug treatment group, n = 3 chips were analyzed and plotted ± SEM for each assay and statistical significance was assessed using a One-Way ANOVA with Tukey’s test (* *p* < 0.05; ** *p* < 0.01; *** *p* < 0.001; **** *p* < 0.0001).

Overall, the CMap predicted drug vorinostat in comparison to the control drugs PGZ and OCA, exhibited complementary effects that mitigated NAFLD progression in the LAMPS. To extend our initial proof-of-concept (PoC) findings, we tested LAMPS models maintained in EMS media containing either control or combinations of pioglitazone (30 µM) and vorinostat (1.7 µM or 5 µM) and monitored the same panel of disease-specific metrics. As shown in Figure 6, while albumin secretion profiles showed no significant differences between vehicle and drug treatment groups, suggesting that these drug combinations did not result in loss of model functionality (Figure 6A), a significant increase in urea nitrogen secretion was observed in both drug combination groups compared to control, suggesting increased model metabolic activity (Figure 6B). In addition, like the LDH profile in Figure 5, there was a significant decrease in LDH secretion (Figure 6C) in the 5 µM vorinostat treatment group, suggesting a reduction in disease-induced cytotoxicity. In contrast to the individual drug testing studies shown in Figure 5, we found an effect on the full complement of disease progression markers measured in this study when pioglitazone and vorinostat were used in combination, as we observed a significant reduction in both lipid accumulation (Figure 6D,E) and stellate cell activation (Figure 6F,G), as well as in the production of the pro-fibrotic markers pro-collagen 1a1 and TIMP-1 (Figure 6H,I) and inflammatory cytokine production (Figure 6J).

### 2.3. Expansion and Complementary Prioritization of CMap Predicted Drugs Using Network Proximity

During the course of these initial studies, the LINCS L1000 database (accessible at clue.io) was significantly expanded, providing an additional 1033 drugs that were annotated in DrugBank and, accordingly, a more comprehensive set of perturbation instances that also encompassed additional cell lines. We took advantage of this larger biological representation by incorporating a percentile statistic for defining an overall CMap score for ranking drugs (Figure 1F,G; Methods and [20]). Using this updated database, many drugs were identified, ranking higher than vorinostat, with the 25 highest shown in Table 1. Some of these drugs having canonical targets associated with NAFLD are predicted to revert 7 of the 12 cluster-based signatures. For example, the NSAID fenoprofen inhibits cyclooxygenase 1 and 2 to modulate prostaglandin synthesis and also activates the peroxisome proliferator receptors, alpha and gamma (PPARα/γ). The androgen receptor agonist oxandrolone, also predicted to revert 7 of the 12 signatures, promoted hepatic ketogenesis in an observational trial of adult males [48] consistent with enhanced fatty acid partitioning from intrahepatic triglycerides towards mitochondrial beta oxidation and 4-hydroxybutyrate formation, as proposed for the reversal of NAFLD resulting from a short-term ketogenic diet [49,50]. Although several of the ranked drugs (Table 1) were structurally steroid-like, considerable structural diversity was evident in the predicted antibiotic and oncology drug classes. The cephalosporin, cefotaxime, interacts with the family of organic anion transporters (OATs or SLC22), whose expression is significantly altered during NAFLD progression [51]. These transporters mediate the hepatic disposition of drugs, xenobiotic metabolites, and endogenous intermediates and metabolites. Targeting NAFLD-associated hepatic proteins that have critical roles both in xenobiotic and endobiotic metabolism may be an emerging theme (see Discussion and [52]) that can be extended to nuclear receptor transcription factors as the diverse drugs tetracycline, SN-38, and the endogenous steroid, pregnanolone, have been shown to interact with PXR [53,54]. In a parallel CMap analysis based on queries derived from 12 patient subtype signatures (complementary to the set of 12 signatures derived from the unsupervised clusters, Table S4; Data files S3–S5), 17/25 of the same predicted drugs (Table 1) were also identified and enriched in the highest ranked drugs.

As a complementary approach to prioritizing the 126 drugs from the CMap analysis (Figure 1G; Table 1 and Table S5), we constructed an NAFLD subnetwork (Figure 1H and Figure S6; Methods) and used proximity to this network [23] as an approach to potentially enhance the specificity and relevance of the CMap analysis. In essence, this algorithm connects NAFLD-associated gene signatures to drug-target profiles and maps the targets of a particular drug to the network protein nodes (Figure 1H–J; Methods). Drugs with target profiles that most closely overlap with a subset of protein nodes in the NAFLD network are prioritized for pharmacological testing in our human liver biomimetic MPS experimental models (Figure 1K and Methods). The KEGG pathway database contains an annotated map of the stage-dependent progression of NAFLD (pathway id: hsa04932, [55,56]). We used this NAFLD progression pathway as an anchor, extending it with 10 interrelated pathways to generate an NAFLD subnetwork in the context of the liver protein–protein interactome (Figure 1H and Figure S6; Methods). From the total number of 9904 DEGs (FDR *p*-value < 0.001) in our three comparisons PLI vs. PN&S, PF vs. PN&S, and PF vs. PLI (Data file S2), 234 DEGs mapped to these 11 NAFLD associated pathways and the background liver PPI network (Figure 1H and Figure S6; Methods). The degrees of the subnetwork nodes range from 0 to 64, with 9.7 neighbors on average for the 234 DEGs, and ranges from 0 to 354, with 52.1 neighbors on average for the background liver network (Data file S6). Among the top 20 hub proteins (Table S7; Data file S6) were HSP90, MAP kinase 8 (MAPK8), NFΚB essential modulator (IKBKG), protein kinase C alpha (PRKCA), caspase 8 (CASP8), signal transducer and activator of transcription 3 (STAT3), mitogen-activated protein kinase kinase kinase 7 (MAP3K7), and protein kinase C zeta type (PRKCZ). 

Among the 126 unique drugs identified by our CMap analysis *per se*, 45 had targets in the liver background network (see Methods). These were further evaluated by determining the network proximity between their targets and the NAFLD subnetwork (Figure S6; Methods) [23]. The network proximity measure for each drug was represented by a z-score ranging from −2.8 to 2.1 (Data file S7; Methods). Negative z-scores indicate that the targets of the drug are more intrinsic to the disease module than a random set of targets. Therefore, the lower the z-score of a predicted drug, the more likely it is to modulate the signaling in the NAFLD disease module. The 25 highest priority drugs and their known targets are shown in Table S7. Among the highest ranked drugs was fenoprofen, also highly ranked by signature frequency (Table 1), bolstering its prioritization for future testing. The HSP90 inhibitor, alvespimycin, was also highly ranked by network proximity, consistent with HSP90 being a critical hub protein in the NAFLD subnetwork (Figure S6; Table S7; Data file S6). ). In addition, a closely related HSP90 inhibitor has been reported to modulate the activation of the NLRP3 inflammasome resulting in efficacy in murine models of NASH [57]. A hallmark of NAFLD is hepatic calcium dyshomeostasis induced by steatosis, which further promotes steatosis, insulin resistance, and ROS that can be ameliorated in murine NASH models by the calcium channel blocker nifedipine [58,59]. Nifedipine and another calcium channel blocker, cinnarizine, were among the drugs ranked higher by network proximity. Two statins, fluvastatin and mevastatin, were also identified by network proximity, consistent with recent meta-analyses [60,61], suggesting the benefit of statin use in NASH development and progression.

**Table 1:** The 25 highest ranked CMap-predicted drugs based on frequency of occurrence across multiple NAFLD-associated gene signature queries. Drugs/small molecules perturbagens identified in more than 1 of the 12 cluster-based gene signature queries were prioritized according to the number of occurrences across the 12 queries and termed: Gene signature-query frequency (Data File S4 and S5). Each signature-based query is indexed s1–12 (see Table S4 and Data file S3) and ordered (from highest to lowest) according to the relative rank of the drug within each query that the drug was identified (i.e., occurrence). Each gene signature-based query is associated with a predominant feature (i.e., disease category) of NAFLD (see Table S4; Data File S3, and Methods). The canonical targets derive from DrugBank (v5.1.4) except for (PXR) (explained in Results). Distinct from Table S5, CMap scores were calculated as percentile scores (see Methods, Results, and [20]), and the 2020 expanded LINCS Database was used as indicated in the Methods and Results. * Denotes compounds also found in a parallel top 25 CMap-predicted drug analysis using clinical classification-based signature queries (Table S4 and Data File S3).

Drug Name (DrugBank ID)	Gene Signature-Query Frequency	Gene Signature Indices (See Table S4) and Their Disease Categorization	Canonical Targets
Eltanolone * (DB12308) (pregnanolone)	7	s5: Insulin Resistance and Oxidative Stress s6: Cell Stress, Apoptosis, and Lipotoxicity s7: Inflammation s3: Inflammation s2: Cell Stress, Apoptosis, and Lipotoxicity s8: Fibrosis s1: Insulin Resistance and Oxidative Stress	(PXR)
Fenoprofen * (DB00573)	7	s5: Insulin Resistance and Oxidative Stress s6: Cell Stress, Apoptosis, and Lipotoxicity s7: Inflammation s8: Fibrosis s2: Cell Stress, Apoptosis, and Lipotoxicity s3: Inflammation s4: Fibrosis	PTGS2, PTGS1, PPARA, PPARG
Oxandrolone * (DB00621)	7	s2: Cell Stress, Apoptosis, and Lipotoxicity s6: Cell Stress, Apoptosis, and Lipotoxicity s3: Inflammation s4: Fibrosis s8: Fibrosis s1: Insulin Resistance and Oxidative Stress s5: Insulin Resistance and Oxidative Stress	AR
Cefotaxime * (DB00493)	6	s2: Cell Stress, Apoptosis, and Lipotoxicity s6: Cell Stress, Apoptosis, and Lipotoxicity s1: Insulin Resistance and Oxidative Stress s7: Inflammation s3: Inflammation s5: Insulin Resistance and Oxidative Stress	SLC22A6, SLC22A8, SLC22A11, SLC22A7, SLC15A1, ALB, SLC15A2
Amorolfine * (DB09056)	5	s3: Inflammation s7: Inflammation s8: Fibrosis s5: Insulin Resistance and Oxidative Stress s6: Cell Stress, Apoptosis, and Lipotoxicity	
Dexamethasone * (DB01234)	5	s3: Inflammation s6: Cell Stress, Apoptosis, and Lipotoxicity s2: Cell Stress, Apoptosis, and Lipotoxicity s7: Inflammation s12: Fibrosis	NR3C1, NR0B1, ANXA1, NOS2, NR1I2 (PXR)
proxyphylline (DB13449)	5	s5: Insulin Resistance and Oxidative Stress s10: Cell Stress, Apoptosis, and Lipotoxicity s11: Inflammation s12: Fibrosis s9: Insulin Resistance and Oxidative Stress	
sn-38 * (DB05482)	5	s4: Fibrosis s2: Cell Stress, Apoptosis, and Lipotoxicity s5: Insulin Resistance and Oxidative Stress s6: Cell Stress, Apoptosis, and Lipotoxicity s7: Inflammation	TOP1, (PXR)
Sulfanitran * (DB11463)	5	s5: Insulin Resistance and Oxidative Stress s6: Cell Stress, Apoptosis, and Lipotoxicity s2: Cell Stress, Apoptosis, and Lipotoxicity s3: Inflammation s1: Insulin Resistance and Oxidative Stress	
Tetracycline * (DB00759)	4	s12: Fibrosis s6: Cell Stress, Apoptosis, and Lipotoxicity s8: Fibrosis s7: Inflammation	PRNP, PADI4, (PXR)
7-hydroxystaurosporine * (DB01933)	4	s8: Fibrosis s6: Cell Stress, Apoptosis, and Lipotoxicity s2: Cell Stress, Apoptosis, and Lipotoxicity s4: Fibrosis	PDPK1
dopamine (DB00988)	4	s12: Fibrosis s9: Insulin Resistance and Oxidative Stress s11: Inflammation s10: Cell Stress, Apoptosis, and Lipotoxicity	DRD2, DRD1, DRD5, DRD3, DRD4, SLC6A3, DBH, HTR1A, HTR7, SLC6A2, SLC6A4, HTR3A, HTR3B, SOD1, SLC18A2
Medrysone * (DB00253)	4	s2: Cell Stress, Apoptosis, and Lipotoxicity s6: Cell Stress, Apoptosis, and Lipotoxicity s5: Insulin Resistance and Oxidative Stress s1: Insulin Resistance and Oxidative Stress	NR3C1
Mestranol * (DB01357)	4	s2: Cell Stress, Apoptosis, and Lipotoxicity s6: Cell Stress, Apoptosis, and Lipotoxicity s4: Fibrosis s7: Inflammation	ESR1
Norethindrone * (DB00717)	4	s10: Cell Stress, Apoptosis, and Lipotoxicity s12: Fibrosis s9: Insulin Resistance and Oxidative Stress s8: Fibrosis	PGR
Troxerutin * (DB13124)	4	s5: Insulin Resistance and Oxidative Stress s8: Fibrosis s7: Inflammation s6: Cell Stress, Apoptosis, and Lipotoxicity	
Brequinar * (DB03523)	3	s7: Inflammation s4: Fibrosis s3: Inflammation	DHODH
bromocriptine (DB01200)	3	s1: Insulin Resistance and Oxidative Stress s11: Inflammation s12: Fibrosis	DRD2, DRD3, HTR1D, ADRA2A, HTR1A, ADRA2C, ADRA2B, HTR2B, DRD4, HTR2A, HTR1B, HTR2C, DRD5, DRD1, ADRA1A, ADRA1B, ADRA1D, HTR7
Cebranopadol * (DB12830)	3	s4: Fibrosis s7: Inflammation s8: Fibrosis	
flucloxacillin (DB00301)	3	s9: Insulin Resistance and Oxidative Stress s11: Inflammation s2: Cell Stress, Apoptosis, and Lipotoxicity	
granisetron (DB00889)	3	s11: Inflammation s10: Cell Stress, Apoptosis, and Lipotoxicity s12: Fibrosis	HTR3A
hexestrol (DB07931)	3	s9: Insulin Resistance and Oxidative Stress s10: Cell Stress, Apoptosis, and Lipotoxicity s11: Inflammation	AKR1C1, ESR1, NR1I2 (PXR), NR1I3
iohexol (DB01362)	3	s1: Insulin Resistance and Oxidative Stress s4: Fibrosis s2: Cell Stress, Apoptosis, and Lipotoxicity	
Melphalan * (DB01042)	3	s3: Inflammation s5: Insulin Resistance and Oxidative Stress s6: Cell Stress, Apoptosis, and Lipotoxicity	
oxacillin (DB00713)	3	s9: Insulin Resistance and Oxidative Stress s12: Fibrosis s11: Inflammation	SLC15A1, SLC15A2

## 3. Discussion

An important outcome of the initial analysis in this study was the identification of differential pathway enrichment profiles among clinically defined stages of NAFLD progression. This information enabled disease states to be defined that could be targeted by systems-based approaches that are more comprehensive and less biased than traditional targeted approaches and, therefore, may be better suited to address the heterogeneity and complex pathophysiology intrinsic to NAFLD. An unsupervised analysis of RNA-seq data from individual liver biopsies derived from a 182 NAFLD patient cohort encompassing a full spectrum of disease progression subtypes from simple steatosis to cirrhosis showed the presence of three patient clusters distinguishable by their pathway enrichment profiles and their predominant association with one of three clinical subtypes: normal/simple steatosis, lobular inflammation, or fibrosis. Pairwise comparisons among these clusters identified differentially enriched pathways consistent with the metabolic underpinning of NAFLD and the pathophysiological processes implicated in its progression that included lipotoxicity, insulin resistance, oxidative and cellular stress, apoptosis, inflammation, and fibrosis. The differentially enriched pathways identified among the pairwise comparisons of clusters originally derived from the unsupervised analysis showed significant congruence with those derived from the clinical subtypes within this patient cohort and, through a meta-analysis, additional patient cohorts. Although from a traditional translational perspective, each of these identified differentially enriched pathways has the potential to be a drug target, their large number and diversity, the prospect of redundancy, and the uncertainty regarding their individual contribution to NAFLD pathogenesis, especially across a heterogeneous patient population, all present challenges to translating this information into therapeutic strategies. The recent failures of several NASH clinical trials due to lack of efficacy [62] are likely the result of this complex pathophysiology emphasizing the need to define and probe therapeutic targets more holistically from the perspective of disease states. 

Guided by systems-based concepts and building upon the gene expression and pathway enrichment analyses, we implemented a QSP approach for defining NAFLD states, predicting drugs that target these states, and testing the predicted drugs in human clinically relevant liver MPS NAFLD models. We defined disease states by first identifying differentially expressed genes for each of the pairwise comparisons among either the three unsupervised cluster groupings or among the three clinically defined clinical groups associated with disease progression. The differentially expressed genes that mapped to differentially enriched pathways were then categorized according to one (or more) of four categories of NAFLD pathophysiological processes in which the pathways are known to participate. This analysis resulted in two sets of twelve gene expression signatures reflecting different states of NAFLD progression. These signatures were then used to query the LINCS L1000 database to identify and rank drugs predicted to revert these gene signatures and, accordingly, normalize their respective corresponding disease states [20,21]. Among the higher CMap-ranked drugs, two complementary criteria, frequency of appearance within each set of 12 signatures or NAFLD subnetwork proximity based on a predicted drug’s known target profile, were used for further prioritization for experimental testing.

To test the predicted drugs in a clinically relevant experimental system, we implemented a human liver acinus MPS, LAMPS, that recapitulates critical structural and functional features of the liver acinus [40,63]. A large and diverse set of biomarkers and image-based analyses measured over time under different media that reflect normal fasting and early and late metabolic syndrome conditions indicated that the human LAMPS also recapitulates critical aspects of NAFLD progression (e.g., simple steatosis, lipotoxicity, oxidative stress, insulin resistance, lobular inflammation, stellate cell activation, and fibrosis) [16,41]. Nevertheless, with the translational goal in mind of identifying disease-modifying therapies, it is important to know if these clinical phenotypes observed pre-clinically arise through those mechanisms that occur in patients. To further establish the clinical relevance of the LAMPS NAFLD model, we implemented a machine learning approach. We trained a transcriptome–based model from the 182 NAFLD cohort representing a full spectrum of disease progression subtypes to classify patients with high specificity. We then implemented this patient-based model consisting of 71 genes, with 57 of these having an independently determined association with NAFLD, to classify the transcriptomes of individual LAMP models treated under media conditions mirroring different stages of disease progression. The congruence between the patient-derived transcriptome-based classification of individual LAMPS and the diverse panel of NAFLD-associated biomarker measurements supports the clinical relevance of the LAMPS as an NAFLD model. Two mechanistically distinct drugs, obeticholic acid and pioglitazone, that have shown some clinical benefit for NAFLD, were then tested as controls, and both exhibited a hepatocellular antisteatotic effect and inhibition of stellate cell activation without an appreciable effect on profibrotic markers. We then tested the top ranked drug from an initial CMap analysis, the HDAC inhibitor vorinostat, predicted to primarily modulate inflammation and fibrosis. Consistent with the NAFLD CMap analysis and in contrast to the control drugs obeticholic acid and pioglitazone, vorinostat showed significant inhibition of proinflammatory and fibrotic biomarkers without an appreciable effect on steatosis. In addition, vorinostat ameliorated disease-induced cytotoxicity. Interestingly, in contrast to our results using vorinostat in the LAMPS model, recent reports show that vorinostat also reduced steatosis [64] and lipid metabolism pathways [65] in rat and HCC monoculture studies, respectively. These contrasting findings demonstrate the variability between animal models and simple cell culture models in assessing drug effects, highlighting the need for implementing all-human MPS model systems in drug testing platforms. Based on the complementary effects exhibited by vorinostat and the control drugs, the combination of vorinostat and pioglitazone was tested and demonstrated significant improvement across the full complement of NAFLD biomarkers. Altogether, these studies provide initial proof-of-concept for a patient-derived QSP platform that can infer disease states from gene expression signatures, predict drugs and drug combinations that can target these disease states, and experimentally test these predictions in clinically relevant NAFLD models.

With the recent expansion of the LINCS L1000 database, we have identified several drugs predicted to be more efficacious than vorinostat for future testing and providing mechanistic inferences. Several of these predicted drugs have known interactions with proteins associated with NAFLD, such as nuclear receptors, and bile and fatty acid transporters. In contrast, others had no known interactions with targets associated with NAFLD despite being predicted to reverse many of the same signatures. These drugs were either highly selective for a particular target such as topoisomerase (e.g., SN-38) or were antibiotics having minimum interactions with human proteins. Further analysis suggested a common thread among many of the predicted drugs that involve nuclear receptors, such as PXR [66] and the related constitutive androstane receptor. PXR is a transcriptional regulator capable of interacting with diverse exogenous and endogenous ligand modulators that have evolved in the liver to have xenobiotic/endobiotic metabolic functions in addition to functions regulating glucose/lipid metabolism/energy, inflammation, and stellate cell activation. Traditional targeted drug discovery approaches have identified FXR and PPAR agonists converging on this broader family of nuclear receptors intimately associated with NAFLD pathophysiology. The QSP approach described here has independently done so in a more comprehensive and unbiased manner with the potential to identify drugs/combinations more efficacious than obeticholic acid and pioglitazone by more completely targeting disease states. In essence, the systems-based platform described here can inform therapeutic strategies that are inherently more pleiotropic than traditional approaches and thus has the potential to address the complexity of transcriptional dysregulation intrinsic to diseases such as NAFLD [12]. The finding that this can be achieved by repurposing approved drugs suggests that acceptable therapeutic indices could result by selectively modulating disease states. In conjunction with the advances in patient-derived iPSC technology [17] and in situ methods for RNA, metabolomic, and proteomic analyses, we anticipate the QSP platform described in this study will become a mainstay for a personalized approach to developing effective NAFLD therapeutic strategies.

## 4. Materials and Methods

### 4.1. Generation of Individual Patient Liver Gene Expression Profiles

The RNAseq data were derived from samples of wedge biopsies taken from the livers of patients undergoing bariatric surgery, as previously described [19]. Patients were diagnosed, and samples were labeled according to the predominant liver histology finding as normal, steatosis, lobular inflammation, or fibrosis [19]. The patient cohort [19] is summarized in Figure 2 and Table S2. The data processing is depicted in the context of the QSP workflow (Figure 1A), and the code used for these analyses can be found at: https://github.com/lefeverde/QSPpaper (accessed on 2 June 2022). Paired fastq-files were pseudo-aligned to the human Ensembl [67] v94 transcriptome using the Kallisto pipeline [68]. The resulting transcript abundances were converted into gene-level estimates using Tximport [69] with the settings recommended for VOOM [70,71]. An exploratory analysis of the gene expression distributions suggested a technical bias exclusive to some of the earliest collected normal and steatotic patient samples that were corrected by the quantile normalization option in the LIMMA-VOOM pipeline [70] (Figure S7A). Principal component analysis (PCA) (Figure S7B) revealed technical heterogeneity (i.e., batch effect) that was accounted for without an appreciable over-correction using surrogate variable analysis [72,73] (Figure S7C). The patient-gene expression matrix encompassing 182 patients and 18,307 genes per patient can be accessed by following the instructions at https://github.com/lefeverde/QSPpaper and served as the primary input for the analyses described below. 

### 4.2. Clustering of Individual Patient KEGG Pathway Enrichment Profiles Associated with NAFLD Clinical Subtypes

The pathophysiology of NAFLD is intrinsically complex and heterogeneous, involving a complex interplay of diverse signaling pathways [2,5]. As an initial step toward understanding the relationship between individual patient pathway enrichment profiles and clinical subtypes, we performed gene set variation analysis (GSVA) [25] (Figure 1B) in conjunction with MSigDB v7.0 C2 KEGG pathways [24]. GSVA, being an intrinsically unsupervised method, enables individual patient pathway enrichment profiles to be generated across a heterogeneous population, providing an advantage over GSEA [74], for example. Importantly and despite the known patient heterogeneity intrinsic to NAFLD, this classification was sufficient to identify and order the three clusters of distinct pathway enrichment profiles with different stages of NAFLD progression and serve as the basis for our subsequent studies in this manuscript. The aforementioned gene expression matrix provided the input for GSVA, resulting in a patient (column) by pathway enrichment (i.e., row of features) matrix. To enable relative comparisons across all identified features, minimizing bias in the ensuing cluster analysis while preserving the presence of outliers within each feature, feature standardization (the mean for each value was subtracted then divided by the standard deviation across each KEGG pathway row) was performed [75]. The pathway enrichment matrix was then subjected to hierarchical clustering (Pearson correlation distance, Ward’s linkage; for details, see https://github.com/lefeverde/QSPpaper), and new groups were identified by cutting the column dendrogram at the 3rd level to create three clusters (Figure 1B and Figure 2). We chose the 3rd level because these clusters had a statistically significant association (Pearson’s Chi-squared Test) with NAFLD clinical subtype (*p* < 2.2 × 10^−16^) and type 2 diabetes (T2D) status (*p* = 0.01). These clusters (Table S2) were named according to the predominant patient sub-classification in each cluster: the first encompassed almost entirely normal and steatosis (PN&S) patients, the second predominantly lobular inflammation (PLI) patients, and the third predominantly fibrosis (PF) patients. Cluster stability was evaluated using the bootstrapping method described in [76]. The 3 identified clusters were compared to new clusters generated from re-sampling using Jaccard coefficients, a metric of similarity between 2 sets [76]. The coefficients were 0.95, 0.62, and 0.72 for PN&S, PLI, and PF, respectively, and above the minimum cutoff of 0.6 proposed by [76]. 

### 4.3. Identification of Differential Gene Expression Signatures for the Three Pairwise Comparisons within the Pathway Enrichment Clusters and within the Clinical Classifications

Having shown an association between the pathway enrichment profiles resulting from unsupervised cluster analysis and the clinical phenotypes (Figure 1B), we next derived two sets of differential gene expression signatures associated with the processes involved in NAFLD development (Figure 1C). One set was derived from the cluster analysis and the other from the clinical classifications. For the former, differentially expressed genes (DEGs) were identified from the aforementioned gene expression data applying the standard LIMMA-VOOM pipeline [70,71] (Figure 1C) for three pairwise comparisons (PLI vs. PN&S, PF vs. PN&S, and PF vs. PLI) (Data file S2). Differentially enriched pathways were identified analogously, except that the GSVA outputs were used (Figure 3; Table S3, and Data file S1). In total, 59, 125, 50 differentially enriched pathways (FDR *p*-value < 0.001) were identified for the 3 pairwise comparisons (Figure 3; Table S3, and Data file S1). A PubMed-directed literature search to assign the differentially enriched pathways into one or more of seven categories (C) (Figure 1D). The first four categories, insulin resistance and oxidative stress (C1); cell stress, apoptosis, and lipotoxicity (C2); inflammation (C3); fibrosis (C4), comprise disease processes strongly associated with NAFLD and constitute our current conceptual framework of NAFLD progression [5]. The three additional categories include: general KEGG-annotated disease-associated pathways (C5); pathways with limited literature association with NAFLD (C6); and pathways with no known association with NAFLD (C7) (Figure 1D). The first four categories (C1–C4) were used for the subsequent generation of NAFLD-associated gene signatures. The gene signatures were created by identifying DEGs (FDR *p*-value < 0.001) that were a component of the [56] differentially enriched pathways (FDR *p*-value < 0.001) associated with the disease processes in categories C1–C4 (Figure 1E; Table S4, and Data file S3) for each of the three pairwise comparisons among the three patient clusters (Figure 2). Four category-specific gene signatures were generated containing the aggregated up- and down-regulated genes for each of these three pairwise comparisons (12 gene signatures in total; Figure 1E; Table S4; and Data file S3). An analogous set of gene signatures was derived from the 3 pairwise clinical classification comparisons lobular inflammation vs. normal and steatosis (Lob vs. N&S), fibrosis vs. normal and steatosis (Fib vs. N&S), and fibrosis vs. lobular inflammation (Fib vs. Lob) Figure 1E; Table S4; Data file S3, for details see https://github.com/lefeverde/QSPpaper.

In sum, two sets of 12 differentially expressed gene signatures were generated, one set derived from distinguishable pathway enrichment profiles associated with different clinical subtypes and the other set derived directly from the clinical classifications (Figure 1E; Table S4 and Data file S3). The differentially expressed genes in each signature reflect pathway dysregulation in NAFLD-associated processes, and the signatures themselves are indicative of a particular disease state at different stages of disease development.

### 4.4. Comparative Pathway Analysis Using Additional NAFLD Patient Datasets

We first performed an internal validation of our pathway results using the 3 pairwise cluster identified patient groupings (PLI vs. PN&S, PF vs. PN&S, and PF vs. PLI) by comparing them to pathway results using 3 pairwise clinical classification comparisons (Lob vs. N&S, Fib vs. N&S, and Fib vs. Lob) (Figure S2). We found that 70–95% of pathways overlapped, and they were all concordant (enriched in the same direction in the cluster grouping and clinical pairwise comparison) (Figure S2). 

We further validated our pathway results (using the cluster groupings) (Figure 1C and Figure 3; Table S3, and Data file S1) by performing concordance analysis on pathway results obtained from re-analyzing 3 external patient microarray datasets: (NASH vs. healthy obese) [77], (NASH vs. simple steatosis) [78], (advanced vs. mild fibrosis) [79] (Figure S3; for details see https://github.com/lefeverde/QSPpaper). This was completed by identifying differentially expressed genes using the standard LIMMA protocol [70,80], then ranking genes by t-statistic and performing GSEA [74] using the MSigDB v7.0 C2 KEGG pathways [24]. In comparison to GSVA, GSEA is better suited to accommodate the smaller number of patient samples per clinical classification in the microarray datasets [77,78,79] and accordingly identified more pathways with small effect sizes as being significant. We compared these differentially enriched (FDR *p*-value < 0.05) pathway results to those in the 182 patient cohort (Figure 1C and Figure 3; Table S3, and Data file S1, [19]), in which a pathway was considered concordant if they were also differentially enriched in the same direction (i.e., up-regulated or down-regulated) in one or more of the microarray cohorts (Figure S3). Conversely, discordance indicates that a pathway is still differentially enriched but in opposite directions (Figure S3). 

### 4.5. Drug Predictions Using the LINCS L1000 Database 

Connectivity mapping (CMap) (15) (Figure 1F) was used to identify drugs and small molecule perturbagens with the potential to normalize the disease state by reverting the aforementioned NAFLD-associated gene signatures (Figure 1E; Table S4 and Data file S3). A pilot study using the two sets of 12 signatures obtained in the previous step was employed to query the LINCS L1000 level 5 (GSE92742, released in 2017) expression database [20] as the initial CMap resource. This database consists of perturbation instances, defined as compound-induced differential gene expression output from a unique combination of cell type, time-point, compound, and compound concentration [20,21]. A subset of the LINCS database with compounds that could be mapped to DrugBank [81] (v5.1.4 used in all analyses) annotations was created by matching the compounds by common name, then by SMILES and/or PubChem ID in cases where the common name differed between databases (see https://github.com/lefeverde/QSPpaper). In total, 1103 DrugBank compounds could be matched to 1495 LINCS compound IDs (there were cases of multiple LINCS compound IDs for the same compound in DrugBank). A LINCS-DrugBank database was generated, comprising a set of 41,710 perturbation instances describing the response to 1103 DrugBank compounds for 70 cell types, at 6 and 24 h time-points, and a range of concentrations. 

During the course of our initial studies, an updated and expanded 2020 LINCS database was released (see clue.io) that we used to generate a 2020 LINCS-DrugBank database see https://github.com/lefeverde/QSPpaper). This version included the 1103 previously matched compounds and an additional 1033 compounds yielding 334,393 instances comprising 2136 DrugBank compounds (2795 LINCS compounds IDs) across 228 cell types, and a range of time-points and concentrations (clue.io). 

The connectivity between each of these drug-induced perturbation instances and each of the 24 input gene signatures was measured by a CMap score (CS) [20,22], composed of two enrichment scores, one for the upregulated genes (ES_up_) and the other for the downregulated genes (ES_down_). The CS was calculated as follows: If the sign of ES_up_ and ES_down_ are the same, CS = 0; otherwise, CS = (ES_up_ − ES_down_)/2 [20]. We obtained results using both the 2017 and 2020 databases (Figure 1F; Data file S4). The former contains 41,710 CSs (one CS for each of the DrugBank perturbation instances), the latter 334,393 CSs (Data file S4). We calculated the *p*-values for the CSs using methods adapted from [82]. For each gene signature, a distribution of random CSs was generated by calculating the CS between a random perturbation instance and random gene set with the same number of up- and down-regulated genes as the gene signature. This was repeated 50,000 times for each gene signature to calculate a *p*-value for each CS. The *p*-values represent the probability of observing the CS using a random set of genes with the same size as the gene signature. The *p*-values were adjusted for multiple testing using the FDR method [83]. 

In order to rank compounds for each of the 24 signature queries, creating a representative CS (i.e., summary statistic) for each compound is necessary since multiple CSs exist for each compound in a single query (Figure 1G). Two approaches were used. The first approach is similar to that used by [84], where the most negative CS (predictive of the compound having the largest effect for inverting the disease gene signature) was chosen for ranking compounds. This approach has the advantage of potentially identifying compounds with maximal efficacy in reversing the gene signature. However, relying on a single or small number of perturbation instances and, therefore, limiting the connection to relevant biological context, may reduce the robustness for translating the CMap predictions to a particular experimental model or clinical cohort. The second approach uses the maximum quantile statistic as described by [20]. The instances are normalized by cell type, then the 33rd (Q_lo_) and 67th (Q_hi_) quantiles of the CSs are computed for each compound, and whichever is larger in magnitude becomes the summary score. If the CSs for a compound are predominantly < 0, then the |Q_lo_| > |Q_hi_| and the summary score is Q_lo_, and vice-versa when the CSs are predominantly > 0 (|Q_lo_| < |Q_hi_| and so the summary score is Q_hi_. The advantage of the maximum quantile approach is that the score is representative of more biological contexts than the single most negative CS approach. 

We initially prioritized the CMap results from the 2017 LINCS database [20] by ranking each drug by the most negative CMap score among all instances for that particular drug, then retaining the top 20 drug predictions from each signature query (Figure 1G, Table S5, and Data file S5). The predictions were further filtered using a threshold of FDR *p*-value < 0.05, and then ranked based on their frequency of appearance across the 12 cluster signatures (Figure 1G, Table S5, and Data file S5). The top 25 compounds from this initial approach are shown in Table S5. We performed a similar approach in a follow-up study using the expanded 2020 LINCS database (accessible at clue.io), except compounds were ranked (in ascending order) using the maximum quantile summary score (described above and in [20]) (Figure 1G, Table 1 and Data file S5). The top 25 predictions from the follow-up study are shown in Table 1.

### 4.6. Drug Prioritization Using Network Proximity Analysis

As a complementary alternative to ranking compounds by frequency of appearance across the signature CMap queries, we adopted the network proximity method as previously described [23]. The method evaluates the distance between the compound’s targets and a given disease module based on the premise that a compound is effective against a disease if its target proteins are within or in the immediate vicinity of the disease module. In essence, this approach provides an independent criterion for selecting from amongst CMap-extracted compounds, to enable further prioritization for experimental testing (Figure 1 Unit 3). 

For determining network proximity, information on a liver-specific PPI network (referred to as the background network) is required. The liver BioSnap network [85] which contains 3180 nodes and 48,409 edges, was retrieved for this aim. A subnetwork from this background network representing the PPIs specific to NAFLD was generated as follows: we selected the KEGG pathway map of NAFLD, which represents a stage-dependent progression of NAFLD (pathway id: hsa04932, [55,56]) in addition to 10 interrelated pathways [55,56]: TNF-signaling (hsa04668), insulin signaling (hsa04910), type II diabetes mellitus (hsa04930), PI3K-Akt signaling (hsa04151), adipocytokine signaling (hsa04920), PPAR signaling (hsa03320), fatty acid biosynthesis (hsa00061), protein processing in the endoplasmic reticulum (hsa04141), oxidative phosphorylation (hsa00190), and apoptosis (hsa04210). We then created an initial subnetwork by taking the intersection of the background network and the genes from these 11 pathways, yielding 390 nodes. We further filtered this initial subnetwork to only include the nodes that were differentially expressed in the three pairwise comparisons (PLI vs. PN&S, PF vs. PN&S, and PF vs. PLI), resulting in a subnetwork of 234 nodes and 1130 edges, termed the NAFLD subnetwork (Figure 1H and Figure S6; Table S7, and Data File S6). 

We performed network proximity (Figure 1I,J) on the results from the cluster signatures queried against the 2020 database prioritized by maximum quantile (Data File S5). The NAFLD subnetwork was used as the disease module to determine the proximity of the 126 CMap prioritized compounds (Data File S5) described above. Among these, 45 are known to target liver-expressed proteins and were subjected to network proximity analysis (Figure 1J, Data file S7) and summarized here. For each drug, we extracted the set of targets (T) from DrugBank v5.1.4 [81], and a distance (d) to the NAFLD subnetwork of 234 nodes (S) was calculated using the liver PPI network as the shortest distance between node t (belonging to T) and the closest node s (belonging to S) averaged over all nodes in T,

$$d\left(S,\;T\right)=\frac{1}{∥T∥}\underset{t\in T}{\sum }\min_{s\in S}d\left(s,\;t\right)$$

A reference distance distribution was constructed, corresponding to the expected distance between two randomly selected groups of proteins of the same size and degree of distribution as the disease proteins and drug targets in the network. This bootstrapping procedure [32] was repeated 1000 times, and the mean (µ) and standard deviation (δ) of the reference distance distribution in conjunction with the distance (*d*) determined above were used to calculate a *z*-score (*d*–µ)/δ for each drug. The *z*-score provides a relative ranking of the drugs vis-à-vis each drug’s potential effects on the NAFLD disease module; a lower *z*-score means a drug’s target profile is closer to the disease module. The resulting top-ranking 25 compounds selected to be prioritized in experiments are presented in Table S8, and the full list of 45 compounds is in Data file S7.

### 4.7. Experimental Drug Testing Using the Human Liver Acinus Microphysiology System (LAMPS)

LAMPS studies (Figure 1K, Figure 5 and Figure 6; Figure S4; Table S6) were carried out as previously described [40,41,63,86] using a single chamber commercial microfluidic device (HAR-V single channel device, SCC-001; Nortis, Inc. Woodinville, WA, USA). LAMPS models were composed of four liver cell types: primary cryopreserved human hepatocytes (HU1960; ThermoFisher, Waltham, MA, USA), primary liver sinusoidal endothelial cells (LSECs; HL160019ECP1; LifeNet Health, Virginia Beach, VA, USA), and THP-1 (Kupffer cell; ATCC, Manassas, VA, USA) and LX-2 (stellate cell; EMD Millipore, Burlington, MA, USA) human cell lines. The percentages of hepatocytes, THP-1, LSEC, and LX-2 cells are consistent with the scaling used in our previously published models [40,41,63,86]. For the drug testing studies described here, LAMPS models were assembled and maintained for 10 days under flow (5 μL/h) in EMS media containing 11.5 mM glucose, 10 nM insulin, 100 μM palmitic acid, and 200 μM oleic acid [41]. LAMPS were maintained for this period in EMS containing (in triplicate for each condition) either vehicle control (0.1% DMSO) or the following drug treatments: 10 μM obeticholic acid (Selleck Chemicals, Houston, TX, USA), 30 μM pioglitazone (Selleck Chemicals), 1.7 μM or 5 μM vorinostat (Selleck Chemicals). For drug combination studies, 30 μM pioglitrazone was combined with either 1.7 μM or 5 μM vorinostat for the duration of the experimental time course. A panel of time course and endpoint NAFLD disease-specific metrics were then examined, including albumin, blood urea nitrogen, and lactate dehydrogenase secretion, lipid accumulation and stellate cell activation, secretion of the pro-fibrogenic markers pro-collagen 1a1 and TIMP-1, and secretion of the cytokines IL-1β, IL-6, IL-8, TNF-α, and MCP-1 [41]. A detailed description for both LAMPS assembly and NAFLD disease progression metrics is provided in the Supplementary Materials and the MPS-Db [41,87].

### 4.8. Performing RNA-seq on the LAMPS NAFLD Models

Separate LAMPS experiments were carried out as described above and previously from our group [41]: LAMPS devices were treated with media mimicking metabolic conditions such as normal fasting (NF), early metabolic syndrome (EMS), and late metabolic syndrome (LMS) (Figure 1L). The LAMPS experiments were carried out at 3-time points: 4, 7, and 10 days with all 3 media conditions (3–4 replicates). Total RNA was extracted from the liver LAMPS chips using Qiazol Reagent (Sigma, St. Louis, MO, USA # R4533) and a 1-Bromo-3 chloropropane (BCP) (Sigma, St. Louis, MO, USA #B9673) phase separation reagent. Further, the aqueous phase of the samples was adsorbed onto Qiagen RNEasy Mini cleanup columns (Qiagen#74204, Germantown, MA, USA) and subjected to DNAse treatment (Qiagen#79254, Germantown, MA, USA) to avoid DNA contamination. Subsequently, the purified RNA was eluted using RNase-free distilled water after washing with 80% ethanol. RNA purity was checked using the nanophotometer (IMPLEN, Westlake, CA, USA). RNA degradation and contamination were monitored on 1% agarose gels. The integrity of RNA was assessed by the RNA Nano 6000 Assay Kitof the Agilent 2100 bioanalyzer (Agilent Technologies, Santa Clara, CA, USA). Samples were required to have a minimum of 200 ng RNA and RIN value greater than 4.0.

A total of 1 μg of total RNA was used as input to each sample preparation. Sequencing libraries were generated using NEBNext Ultra RNA Library Prep Kit for Illumina (Ipswitch, MA, USA). Briefly, mRNA was purified from total RNA using poly-T oligo-attached magnetic beads. Fragmentation was carried out using divalent cations under elevated temperature in NEB Next First Strand Synthesis Reaction Buffer (5X). First strand cDNA was synthesized using random hexamer primer and M-MuLV Reverse Transcriptase (RNase H-). Second strand cDNA synthesis was subsequently performed using DNA Polymerase I and RNase H. Remaining overhangs were converted into blunt ends via exonuclease/polymerase activities. After adenylation of 3′ ends of DNA fragments, NEB Next Adaptor with hairpin loop structure was ligated to prepare for hybridization. To select cDNA fragments of preferentially 150~200 bp in length, the library fragments were purified with AMPure XP system (Beckman Coulter, Beverly, CA, USA). Then, 3 μL USER Enzyme (NEB, USA) was used with size-selected, adaptor-ligated cDNA at 37 °C for 15 min, followed by 5 min at 95 °C before PCR. Then, PCR was performed with Phusion High-Fidelity DNA polymerase, Universal PCR primers, and Index (X) Primer. Then, PCR products were purified (AMPure XP system), and library quality was assessed on the Agilent Bioanalyzer 2100 system. Samples were required to have a cDNA library concentration > 0.5 ng/microL, a single qPCR peak at 2–30 nM, no adapter contaminations or primer dimers, and product size of 350–520 bp. 

The clustering of the index-coded samples was performed on a cBot Cluster Generation System using PE Cluster Kit cBot-HS (Illumina) according to the manufacturer’s instructions. After cluster generation, the library preparations were sequenced on an Illumina platform, and 125 bp/150 bp paired-end reads were generated.

### 4.9. Concordance Analysis of Differentially Enriched Pathways in Patients and LAMPS 

The raw LAMPS transcriptome data (accessible following the instructions at https://github.com/lefeverde/QSPpaper) were processed using the same pipeline as described for the patients (Figure 1L). Differentially expressed genes were identified using the standard LIMMA-VOOM protocol [70,71] in which the genes were fit with a linear model for media treatment and timepoint. As we are interested in the treatment effects, time point was treated here as a confounding variable [88]. We identified differentially expressed genes (Data file S8) for LAMPS by performing three pairwise comparisons consisting of EMS vs. NF, LMS vs. NF, and LMS vs. EMS, which are meant to be analogous to the patient pairwise comparisons (Lob vs. N&S, Fib vs. N&S, and Fib vs. Lob). The phenotypes of NF, EMS, and LMS range from minimal, moderate, and pronounced levels of steatosis, inflammation, and fibrosis, respectively [41] (Figure 4B). Differentially enriched pathways were identified by ranking the genes by *t*-statistic for each pairwise comparison and then performing GSEA [74] using the MSigDB v7.0 C2 KEGG pathways [24] for both the LAMPS and patient comparisons (Figure 1L and Figure S5; Data file S9).

Using this differential enrichment pathway analysis as input, we performed a concordance analysis of the LAMPS and matched patient pairwise comparisons (Figure 1L and Figure S5). A pathway was considered concordant if it was significantly (FDR *p*-value < 0.05) regulated in the same direction in both the LAMPS and matched patient pairwise comparisons (Figure S5). Conversely, discordance indicates that a differentially enriched pathway identified in both comparisons is regulated in opposite directions. 

### 4.10. Comparing LAMPS NAFLD Model Transcriptomes to Patients via Multinomial Logistic Regression with Elastic Net Penalization (MLENet) 

We used an MLENet model [89] to compare the LAMPS to patients since this is a classifier that performs feature (i.e., gene) selection (Figure 1L; for details, see https://github.com/lefeverde/QSPpaper). The patient gene expression data (accessible following the instructions at https://github.com/lefeverde/QSPpaper) was prepared by first ranking the genes by variance and taking the top 7500 (this is done to reduce overfitting by removing uninformative features). The same variance thresholding was applied to the LAMPS expression matrix (see https://github.com/lefeverde/QSPpaper). Next, genes that were not in both the variance filtered LAMPS and patient expression matrixes were removed from both, yielding a set of 4057 genes. For the LAMPS gene expression matrix, we used surrogate variable analysis [72,73] to predict and then remove unwanted sources of variation (timepoint and possible cell ratio differences). Both the patient and LAMPS matrixes were standardized (gene-wise) to have zero mean and unit variance. 

We used a nested cross-validation approach to ensure that MLENet could successfully differentiate between the 4 patient histological classifications (normal, steatosis, lobular inflammation, or fibrosis). To do this, we used the Glmnet package [89] applying the appropriate distribution (multinomial) and setting (alpha = 0.95) that in initial trials enabled optimal performance for classifying the LAMPS samples. The nested cross-validation was performed by first generating 100 sets of training and test data (Figure 4A). This was completed by sampling 70% of the patient from each class to create a training subset and then using the remaining 30% for the testing subset (Figure 4A). For each of the 100 sets, we trained an MLENet model on the training subset using *cv.glmnet* [89] and then used the testing subset to evaluate the model’s performance by calculating the specificity and sensitivity of the 4 patient classes (Figure 4A).

After ensuring that the MLENet approach could accurately classify patients with a mean (numbers in parenthesis are standard deviation) specificity of 0.93 (0.03), 0.83 (0.03), 0.98 (0.02), 0.95 (0.03) for normal, steatosis, lobular inflammation, and fibrosis, respectively, we trained a final model using the 182 patients using the parameters described above (Figure 1L and Figure 4B). The final MLENet model selected 71 genes, of which, the majority (80%) had prior association with NAFLD in independent studies (usually being differentially expressed in other studies, see Data file S10). We used this final MLENet model to classify the LAMPS samples as belonging to one of the 4 patient classes (Figure 4B; for details, see https://github.com/lefeverde/QSPpaper).

## Data Availability

The article includes all data generated or analyzed during the study. Drug study experimental data are available through the University of Pittsburgh Drug Discovery Institute Microphysiology Systems Database (https://mps.csb.pitt.edu/). Data from experimental studies performed in this work are available at: https://mps.csb.pitt.edu/assays/assaystudyset/27/. The analysis code generated during the study is available at: https://github.com/lefeverde/QSPpaper. The raw data is available at: https://pitt-my.sharepoint.com/:u:/g/personal/del53_pitt_edu/ERbd6dXUY9pMq8UMPzLLEXoBYkr0PpWyDJgesAzsad5KBg?e=ZXmav3 (accessed on 2 June 2022).

## Supplementary Materials

The following supporting information can be downloaded at: https://www.mdpi.com/article/10.3390/metabo12060528/s1, Figure S1: Distribution of differentially enriched pathways and their respective KEGG groups and NAFLD categories of pairwise comparisons performed using the patient clinical classifications (complements Figure 3); Figure S2. Venn diagrams showing the overlap of differentially enriched pathways (FDR *p*-value < 0.001) identified in the cluster (left circle) and clinical label (right circle) pairwise comparisons (Supports Figure 3 and Figure S1); Figure S3: Concordance analysis of the differentially enriched pathways in the cluster pairwise comparisons (left circle) and pathway list derived from microarray datasets (right circle); Figure S4: Using the Biomimetic Human Liver Acinus MicroPhysiology System (LAMPS) for proof-of-concept experimental testing of CMap-predicted drugs; Figure S5: Concordance analysis of the differentially enriched pathways in the LAMPS (left circle) and phenotypically matched patient pairwise comparisons; Figure S6: NAFLD associated protein interactome; Figure S7: Exploratory data analysis and PCA of the patient transcriptome; Table S1: Index of associated tables, figures, data files, or notebook analyses for each step in Figure 1; Table S2: Distribution of NAFLD patient subtypes within the three clusters defined in Figure 2; Table S3: The differentially enriched pathways across 7 NAFLD categories for each pairwise cluster and clinical classification comparison (supporting Figure 3 and Figures S1); Table S4. Gene signature index (created using Data file S3); Table S5: Twenty-five highest ranked predicted drugs based on initial CMap analysis; Table S6: Drug binding and cytotoxicity profiles for compounds used in LAMPS studies; Table S7: The 20 highest ranked hubs (proteins/targets) by degree in the NAFLD subnetwork; Table S8. Prioritization of CMap-predicted drugs and small-molecule perturbagens based on NAFLD subnetwork proximity; Data file S1. Differentially enriched pathways for each pairwise cluster and clinical classification comparison; Data file S2. DEGs resulting for each pairwise cluster and clinical classification comparisons; Data file S3: Gene signatures used for CMap analysis; Data file S4: CMAP scores of small molecules with a DrugBank ID for the 24 queries described in the Methods; Data file S5: List of top 20 CMap predictions from both the 2017 and 2020 LINCS databases and both ranking methods (“Best score” and “Percentile score”) from the 24 signatures; Data file S6. Degree of the nodes in the NAFLD subnetwork (Figure S5); Data file S7. Network proximity-determined Z-scores for the highest ranking CMap-predicted drugs with targets mapping to the NAFLD subnetwork; Data file S8. DEGs resulting from the LAMPS pairwise comparisons (EMS vs. NF, LMS vs. NF, LMS vs. EMS); Data file S9. Differentially enriched pathways the LAMPS pairwise comparisons (EMS vs. NF, LMS vs. NF, LMS vs. EMS); Data file S10. The 71 features selected by the final MLENet model; Expanded methods. Reference [90] are cited in the supplementary materials.
