# Supplementary material for: A Quantitative Systems Pharmacology Platform Reveals NAFLD Pathophysiological States and Targeting Strategies
Source: Metabolites. 2022 Jun 7;12(6):528. doi: 10.3390/metabo12060528 (PMC9227696; doi:10.3390/metabo12060528)
Supplement: Supplementary file 1 [file metabolites-12-00528-s001.zip › metabolites-1726303-supplementary.pdf]

# Supplementary material

# A Quantitative Systems Pharmacology Platform Reveals NAFLD Pathophysiological States and Targeting Strategies

**Daniel E. Lefever[1*], Mark T. Miedel[1,2*], Fen Pei[2*], Johanna K. DiStefano[3], Richard Debiasio[1], Tong Ying Shun[1], Manush Saydmohammed[1], Maria Chikina[2,5], Lawrence A. Vernetti[1,2], Alejandro Soto-Gutierrez[1,4,5,6], Satdarshan P. Monga[1,4,5], Ramón Bataller[7], Jaideep Behari[7,8], Vijay K. Yechoor[1,9], Ivet Bahar[1,2], Albert Gough[1,2], Andrew M. Stern[1,2†], D. Lansing Taylor [1,2,5†]**

[1]Drug Discovery Institute, University of Pittsburgh; Pittsburgh, PA, 15261, USA.
[2]Department of Computational and Systems Biology, University of Pittsburgh School of Medicine; Pittsburgh, PA, 15260, USA.
[3]Diabetes and Fibrotic Disease Unit, Translational Genomics Research Institute TGen; Phoenix, AZ, 85004, USA.
[4]Department of Pathology, University of Pittsburgh School of Medicine; Pittsburgh, PA, 15261, USA.
[5]Pittsburgh Liver Research Center, University of Pittsburgh; Pittsburgh, PA, 15261, USA.
[6]McGowan Institute for Regenerative Medicine, University of Pittsburgh; Pittsburgh, PA, 15203, USA.
[7]Department of Medicine, Division of Gastroenterology Hepatology and Nutrition, University of Pittsburgh School of Medicine; Pittsburgh, PA, 15261, USA.
[8]UPMC liver clinic, University of Pittsburgh Medical Center; Pittsburgh, PA, 15213, USA.
[9]Department of Medicine, Division of Endocrinology and Metabolism, University of Pittsburgh School of Medicine; Pittsburgh, PA, 15203, USA.

[*]Co-First Authors
[†]Co-corresponding Authors: D. Lansing Taylor dltaylor@pitt.edu, and Andrew M. Stern sternam@pitt.edu

Table of Contents

*Figure S1. Distribution of differentially enriched pathways and their respective KEGG groups and NAFLD categories of pairwise comparisons performed using the patient clinical classifications (complements Figure 3).*

*Figure S2. Venn diagrams showing the overlap of differentially enriched pathways (FDR p-value < .001) identified in the cluster (left circle) and clinical label (right circle) pairwise comparisons (Supports Figures 3 & S1).*

*Figure S3. Concordance analysis of the differentially enriched pathways in the cluster pairwise comparisons (left circle) and pathway list derived from microarray datasets (right circle).*

*Figure S4. Using the Biomimetic Human Liver Acinus MicroPhysiology System (LAMPS) for proof-of-concept experimental testing of CMap-predicted drugs.*

*Figure S5. Concordance analysis of the differentially enriched pathways in the LAMPS (left circle) and phenotypically matched patient pairwise comparisons.*

*Figure S6. NAFLD associated protein interactome (link to file).*

*Figure S7. Exploratory data analysis and PCA of the patient transcriptome.*

*Table S1. Index of associated tables, figures, data files or notebook analyses for each step in Figure 1.*

*Table S2. Distribution of NAFLD patient subtypes within the three clusters defined in Figure 2.*

*Table S3. The differentially enriched pathways across 7 NAFLD categories for each pairwise cluster and clinical classification comparison (supporting Figures 3; S1-S3 ) (Link to excel file).*

*Table S4. Gene signature index (created using Data file S3).*

*Table S5. 25 highest ranked predicted drugs based on initial CMap analysis.*

*Table S6. Drug binding and cytotoxicity profiles for compounds used in LAMPS studies.*

*Table S7. The 20 highest ranked hubs (proteins/targets) by degree in the NAFLD subnetwork.*

*Table S8. Prioritization of CMap-predicted drugs and small-molecule perturbagens based on NAFLD subnetwork proximity.*

*Data file S1. Differentially enriched pathways for each pairwise cluster and clinical classification comparison (Link to csv file).*

*Data file S2. DEGs resulting for each pairwise cluster and clinical classification comparisons (Link to csv file).*

*Data file S3. Gene signatures used for CMap analysis (Link to csv file).*

*Data file S4. CMAP scores of small molecules with a DrugBank ID for the 24 queries described in the Methods (Link to csv file).*

***Data file S5. List of top 20 CMap predictions from both the 2017 & 2020 LINCS databases and both ranking methods ("Best score" and "Percentile score") from the 24 signatures (link to file).***

***Data file S6. Degree of the nodes in the NAFLD subnetwork (Figure S5, Link to csv file).***

***Data file S7. Network proximity determined Z-scores for the highest ranking CMap-predicted drugs with targets mapping to the NAFLD subnetwork (Link to csv file).***

***Data file S8. DEGs resulting from the LAMPS pairwise comparisons (EMS vs NF, LMS vs NF, LMS vs EMS) (Link to csv file).***

***Data file S9. Differentially enriched pathways the LAMPS pairwise comparisons (EMS vs NF, LMS vs NF, LMS vs EMS) (Link to csv file).***

***Data file S10. The 71 features selected by the final MLENet model (Link to csv file).***

***Expanded Methods***

***References***

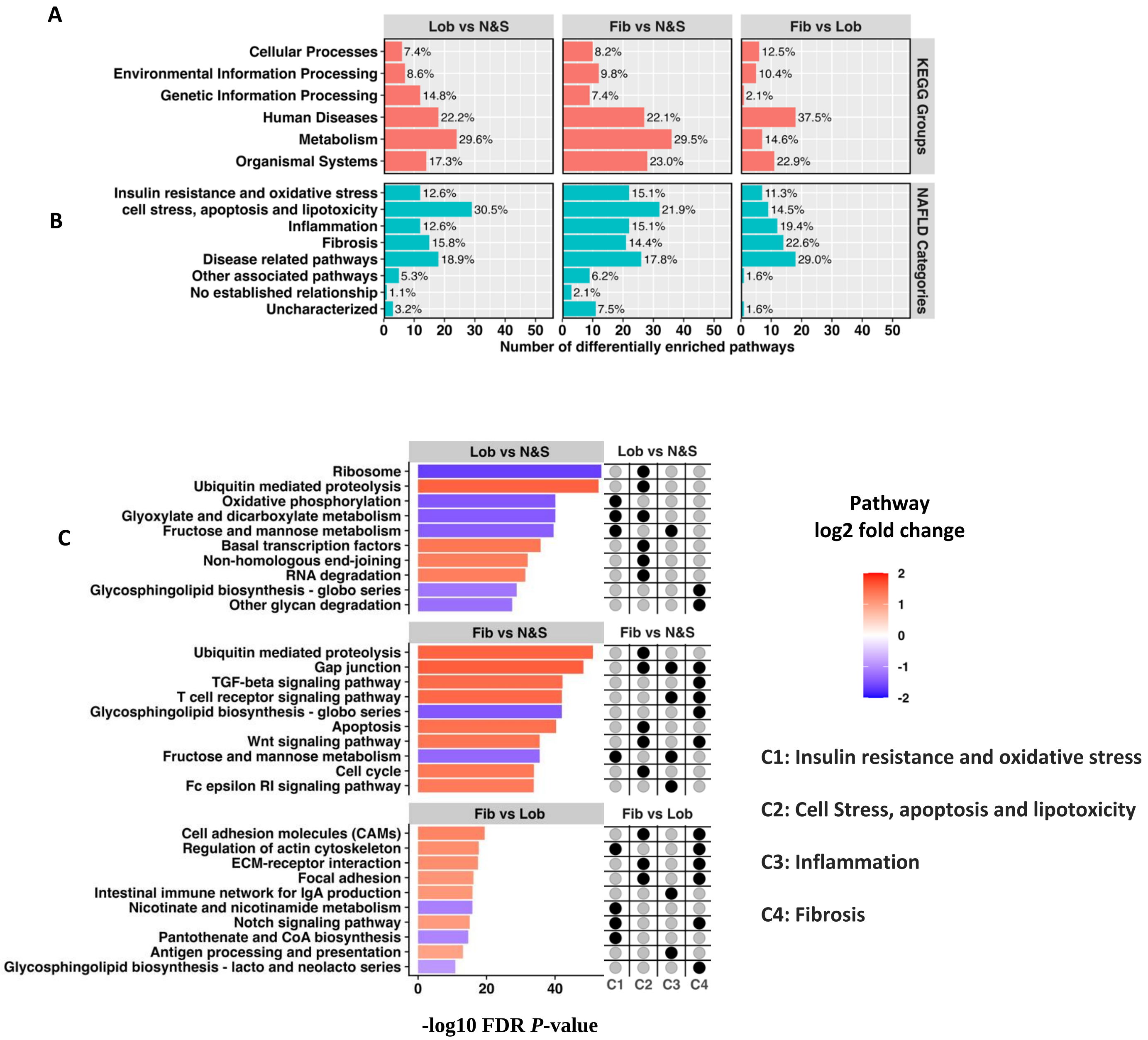


**Figure S1. Distribution of differentially enriched pathways and their respective KEGG groups and NAFLD categories of pairwise comparisons performed using the patient clinical classifications (complements Figure 3).**

The number of differentially enriched pathways identified between the Lobular inflammation vs Normal & Steatosis (Lob vs N&S), Fibrosis vs Normal & Steatosis (Fib vs N&S), and Fibrosis vs Lobular inflammation (Fib vs Lob), pairwise comparisons were 81, 122, and 48, respectively (adj. *p*-value <0.001). Their distribution (and percent contribution) with respect to KEGG Groups (**A**) and NAFLD categories (**B**) are detailed in **Table S3** and **Data file S1**. The top ten differentially enriched pathways for each comparison (ranked by the FDR adjusted *p*-values through the linear modelling equivalent of a two sample, moderated t-test) are shown along with their association (black circles) with NAFLD categories C1-4 (as indicated and defined in the Main Text) (**C**). The colors of the bars represent the directionality and relative enrichment of each pathway for each of the pairwise comparisons.

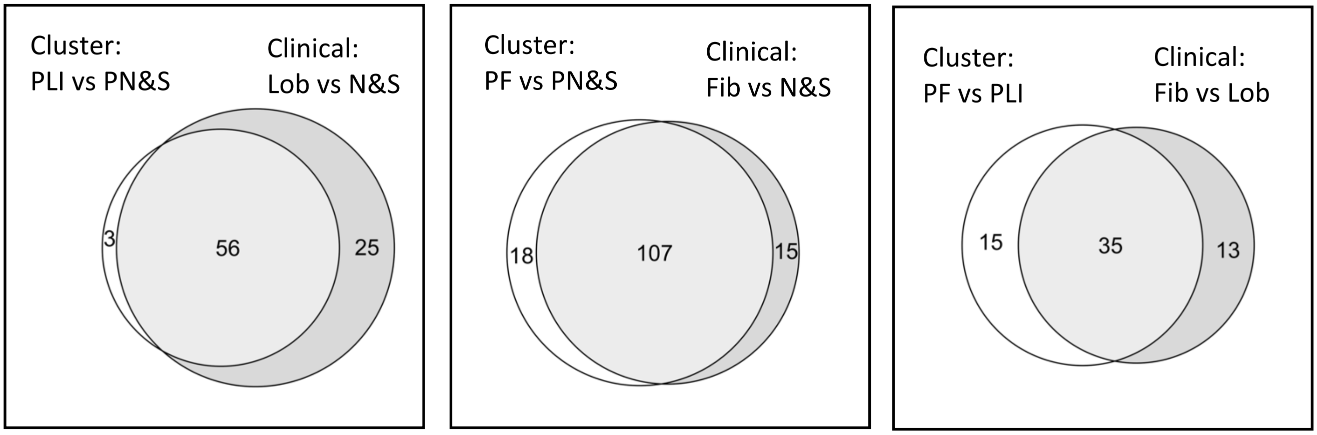


**Figure S2. Venn diagrams showing the overlap of differentially enriched pathways (FDR *p*-value < .001) identified in the cluster (left circle) and clinical label (right circle) pairwise comparisons (Supports Figures 3 & S1).**

Differentially enriched pathways (**Table S3** & **Data file S1**) were identified using the GSVA-limma-voom approach described in the Methods. All of the overlapping pathways were concordant.

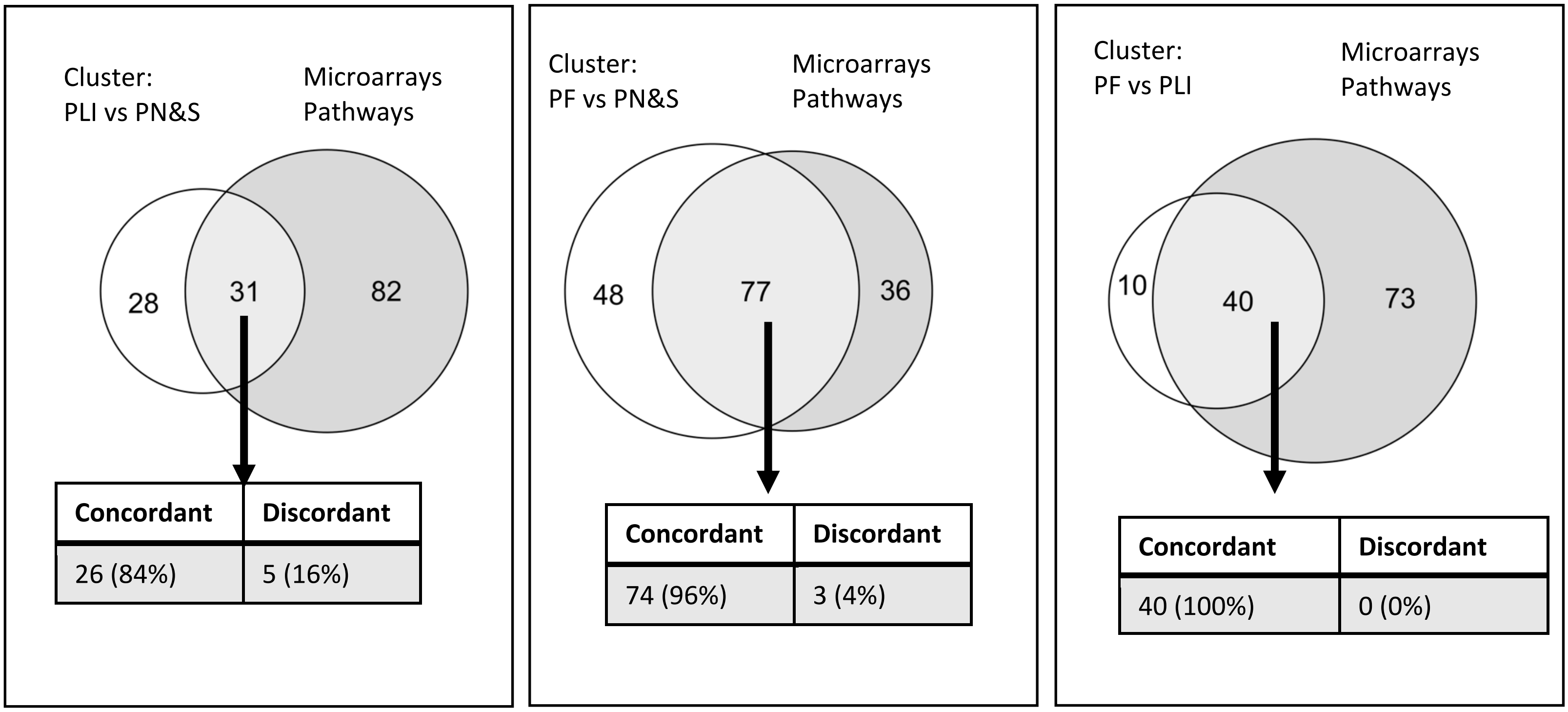


**Figure S3. Concordance analysis of the differentially enriched pathways in the cluster pairwise comparisons (left circle) and pathway list derived from microarray datasets (right circle).**

The microarray pathway list is the combined differentially enriched pathways found from re-analyzing the following datasets (the specific pairwise comparisons are indicated in the parenthesis): Ahrens et al., (*9*) (NASH vs healthy obese), Arendt et al., (*10*) (NASH vs simple steatosis), Murphy et al., (*11*) (Advanced vs mild fibrosis). See **Methods** and https://github.com/lefeverde/QSPpaper for details. Differentially enriched (FDR *p*-value < 0.05) pathways in the 182 patient cohort were considered concordant if they were also differentially enriched in the same direction (i.e., up-regulated or down-regulated) in one or more of the microarray cohorts. Conversely, discordance indicates that a pathway is still differentially enriched but in opposite directions.
**$p$-value <= .004 (Exact Binomial Test, % is estimated effect size)

A.

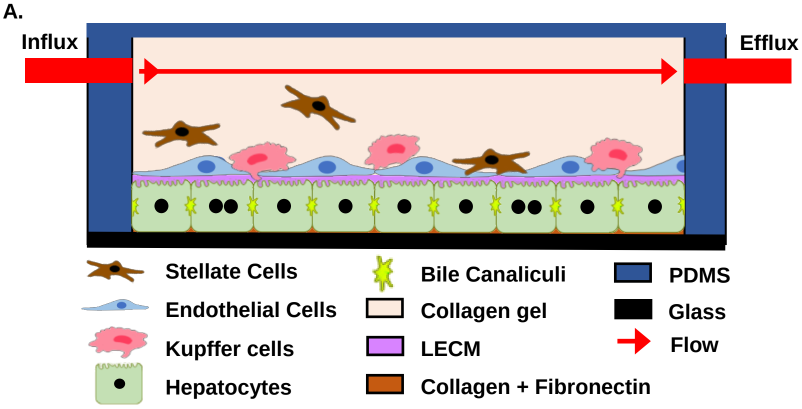


B.

**LAMPS Metrics:**

- Albumin, BUN, LDH Efflux
- Hepatic Steatosis (LipidTOX™)
- Stellate Cell Activation (α-SMA)
- Immune Activation Cytokine Panel (TNF-α, IL-6, IL-8, IL-1β, MCP-1)
- Fibrosis Markers (Pro-Col 1a1, TIMP-1 Efflux)

**Figure S4. Using the Biomimetic Human Liver Acinus MicroPhysiology System (LAMPS) for proof-of-concept experimental testing of CMap-predicted drugs.**

**A)** Diagram illustrating the typical cell organization in the LAMPS model after cell seeding. The LAMPS is a polydimethylsiloxane (PDMS)-based all-human single flow channel microfluidic cell platform designed to partially recapitulate the structure and functions of the human liver acinus (*12-16*). Primary hepatocytes are first seeded on a layer of collagen and fibronectin. Primary liver sinusoidal endothelial cells (LSEC) and Kupffer-like THP-1 cells are seeded 18-24 h after hepatocytes on a layer of decellularized porcine liver extracellular matrix (LECM). Following this, the LX-2 stellate cell line is seeded last, settling in collagen that fills the chamber. The direction of media flow is indicated by the arrow. A detailed description of the model setup can be found in the Supplementary Materials section. **(B)** We have recently demonstrated that this model system recapitulates key aspects of NAFLD progression using media containing key NAFLD drivers including increased levels of glucose, insulin and free fatty acids (*16, 17*). Using this platform, we examined a panel of metrics to monitor NAFLD disease-specific phenotypes, in the presence of CMap-predicted drugs, including model functionality (albumin and blood urea nitrogen (BUN) secretion) and cytotoxicity (lactate dehydrogenase secretion), hepatocellular steatosis (LipidTOX™ labeling), stellate cell activation [α-smooth muscle actin (SMA) antibody staining], and the production of a panel of pro-inflammatory cytokines (TNF-α , IL-6, IL-8, IL-1β and MCP-1) and fibrotic markers (Pro-collagen 1A1 and TIMP-1).

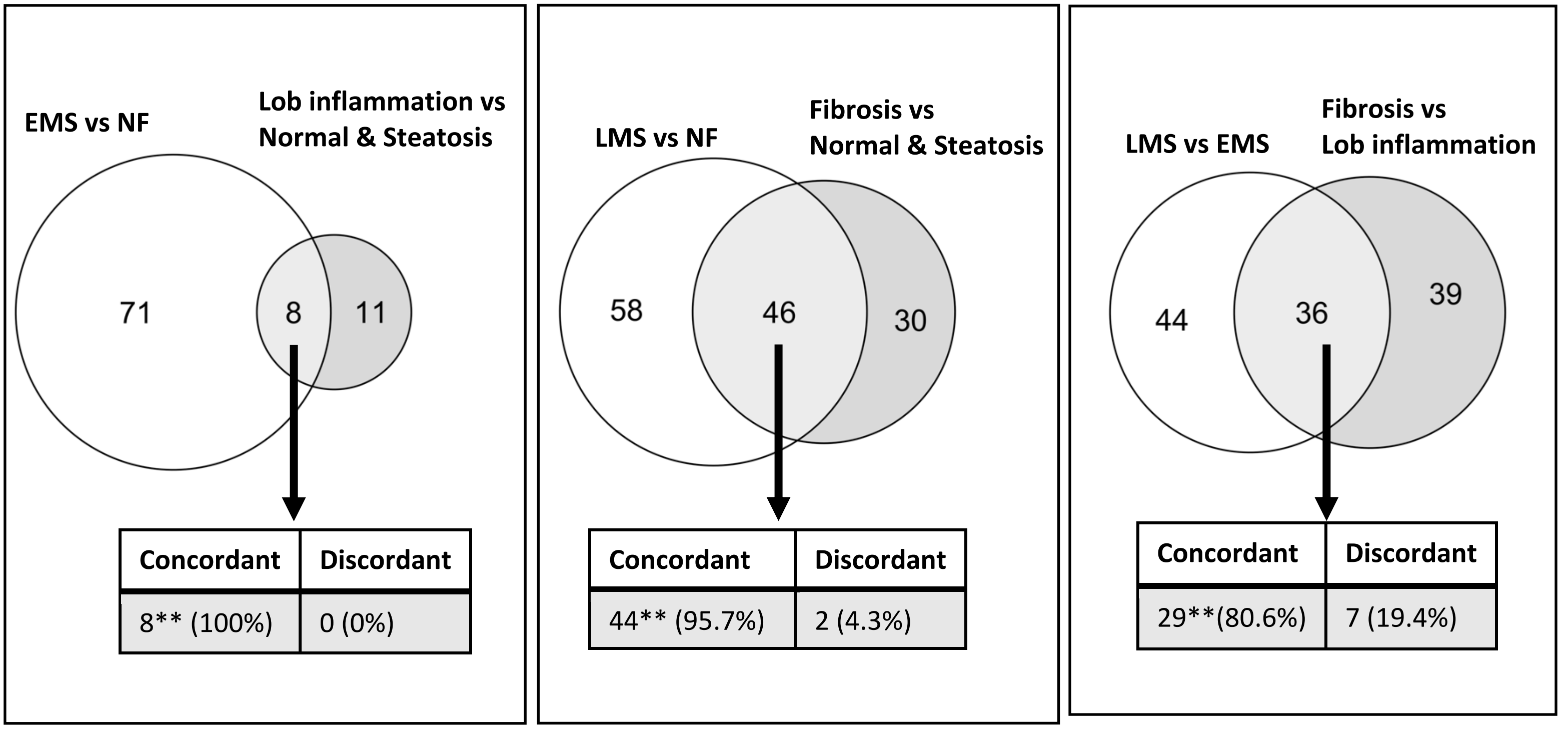


**Figure S5. Concordance analysis of the differentially enriched pathways in the LAMPS (left circle) and phenotypically matched patient pairwise comparisons.**
The pathways were identified using GSEA as described in the Methods for the pairwise comparisons. A pathway was considered concordant if it was significantly regulated (FDR *p*-value < .05) in the same direction (up/down) in the LAMPS and patient comparisons, discordance is when pathways are differentially expressed but have opposite signs.

***p*-value <= .004 (Exact Binomial Test, % is estimated effect size)

**Figure S6. NAFLD associated protein interactome (link to file).**

A subnetwork of the human liver protein interactome involving NAFLD associated protein-protein interactions. The indicated nodes represent those proteins encoded by the DEGs among the pairwise comparisons for the three clusters defined in **Figure 2**. The degrees of these nodes are shown in **Data file S6** and the 20 hubs with the highest degrees are shown in **Table S7**.

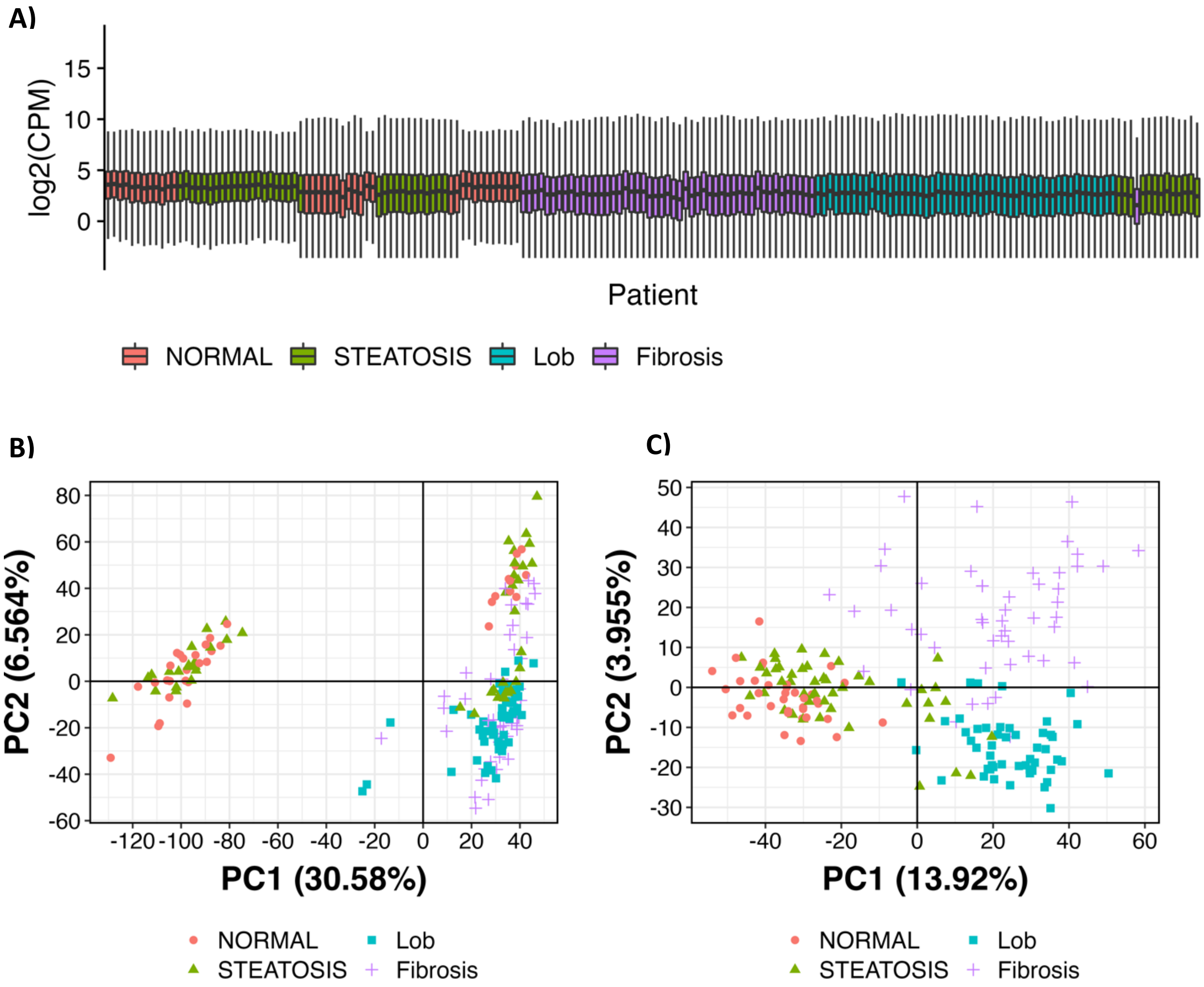


**Figure S7. Exploratory data analysis and PCA of the patient transcriptome.**

**A)** Shows the boxplots (outliers are not shown) of the log2 transformed counts per million log2(CPM) gene expression values for each patient, ordered by the patient ID (i.e., the order the samples were processed). The distributions of normal and steatosis patients tend vary in discrete blocks of samples in contrast to lobular inflammation, fibrosis, or a set of steatosis patients collected later on in the experiment. This suggests the presence of a technical artifact which affects the distribution that is confounded with the patient classifications. Hence, we used quantile normalization to correct for this effect. Principal component analysis (PCA) of the log2(CPM) gene expression values revealed the presence of a batch effect (**B**). We therefore used surrogate variable analysis (SVA) to estimate covariates that could account for this unwanted heterogeneity while still retaining the biological variation. **C)** Shows the PCA plot using the SVA corrected gene expression matrix.

| | | | Output | | |
|---|---|---|---|---|---|
| **Unit** | **Step** | **Input data** | **Figures** | **Tables** | **Data files** |
| **1** | **A. Gene expression profiles of patient samples across different NAFLD subtypes** | **Ensembl v94 Zerbino et al., 2017 (*1*)**<br>**Transcriptome data Gerhard et al., 2018 (*2*)** | **Figure S7** | | **Analysis notebook Lefever, 2021 (*3*)** |
| | **B. Clustering using individual patient pathway enrichment profiling** | **KEGG MSigDB v7.0 Liberzon et al., 2011 (*4*)**<br>**Step A** | **Figure 2** | **Table S2** | **Analysis notebook Lefever, 2021 (*3*)** |
| | **C. Identification of DEGs and differentially enriched pathways** | **Steps A,B** | **Figures 3; S1-S2** | **Table S3** | **Data files S1-S2** |
| | **D. Categorization of differentially enriched pathways based on NAFLD progression** | **Step C** | **Figures 3; S1-S2** | **Table S3** | **Data file S1** |
| **2** | **E. Generating gene signatures based on DEGs/differentially enriched pathways from each category of each comparison** | **Steps C,D** | | **Table S4** | **Data file S3** |
| | **F. Predicting drugs using CMAP by screening L1000 database** | **LINCS L1000 Subramanian et al., 2017 (*5*)**<br>**Step E** | | | **Data file S4** |
| | **G. Prioritizing drugs based on signature frequency and rank** | **Step F** | | **Table 1; S5** | **Data file S5** |
| **3** | **H. Generating NAFLD related subnetwork based on KEGG pathways and liver PPIs** | **BioSnap Marinka et al., 2018 (*6*)**<br>**Step C** | **Figure S6** | **Table S7** | **Data file S6** |
| | **I. Identification of targets for the top ranked drugs** | **DrugBank v5.4.1 Wishart et al., 2018 (*7*)**<br>**Step H** | | | **Analysis notebook Lefever, 2021 (*3*)** |
| | **J. Prioritizing the predicted drugs with Network Proximity** | **Step I** | | **Table S8** | **Data file S7** |
| **4** | **K. Testing predicted drugs in a human liver MPS model** | **Steps G, J** | **Figures 5-6; S4** | **Table S6** | |

| | | | | | |
|---|---|---|---|---|---|
| | **L. Establish clinical relevance of human liver MPS model** | **Step A**<br>**LAMPS transcriptome data** | **Figures 4; S5** | | **Data file S8-S10** |

**Table S1. Index of associated tables, figures, data files or notebook analyses for each step in Figure 1.**

| Clinical diagnosis | Normal | Steatosis (Grade) | | Lobular Inflammation (Score) | | Fibrosis (Score) | | | Patients per cluster |
|---|---|---|---|---|---|---|---|---|---|
| Cluster | | 2 | 3 | 1 | 2 | 3 | 3.5 | 4 | |
| PN&S | 35 (9) | 26 (8) | 12 (7) | 3 (1) | 1 (0) | 1 (0) | 1 (0) | 0 (0) | 79 (25) |
| PLI | 0 (0) | 2 (0) | 1 (0) | 23 (9) | 3 (0) | 1 (0) | 3 (1) | 4 (2) | 37 (12) |
| PF | 1 (0) | 4 (1) | 1 (0) | 11 (2) | 9 (2) | 15 (12) | 11 (9) | 14 (10) | 66 (36) |
| total | 36 (9) | 32 (9) | 14 (7) | 37 (12) | 13 (2) | 17 (12) | 15 (10) | 18 (12) | 182 (73) |

**Table S2. Distribution of NAFLD patient subtypes within the three clusters defined in Figure 2.**

The numbers of patients diagnosed with type 2 diabetes (T2D) are indicated in parentheses in each case. The clusters of the cohort samples are significantly associated (Pearson's Chi-squared test) with NAFLD subtype ($p < 2.2e-16$) and T2D status ($p = 0.01$). The *red* values denote the predominant clinical subtype within each cluster**.**

**Table S3. The differentially enriched pathways across 7 NAFLD categories for each pairwise cluster and clinical classification comparison (supporting Figures 3; S1-S3 ) (Link to excel file).**

**Data file S1** was used to create these tables. The excel file consists of 6 sheets: PLI vs. PN&S, PF vs. PN&S, PF vs. PLI, Lob vs N&S, Fib vs N&S, and Fib vs Lob comparisons. The columns of the tables are as follows:

- KEGG Pathway name and ID
- KEGG pathway group
- KEGG pathway subgroup
- NAFLD categorization of KEGG pathway (see **Methods**)
    - C1: Insulin resistance and oxidative stress
    - C2: cell stress, apoptosis and lipotoxicity
    - C3: Inflammation
    - C4: Fibrosis
    - C5: Disease related pathways
    - C6: Other associated pathways
    - C7: No established relationship
- log2 Fold change: estimate of the log2-fold-change of the comparison
- FDR corrected *p*-value: False discovery rate
- PMIDs: The PMIDs for the references which support the NAFLD categorization

| NAFLD pathway category | Cluster gene signature ID | Cluster comparison | Clinical gene signature ID | Clinical Comparison |
|---|---|---|---|---|
| C1: Insulin resistance and oxidative stress | s1 | PLI vs. PN&S | s*1 | Lob vs N&S |
| C2: cell stress, apoptosis and lipotoxicity | s2 | PLI vs. PN&S | s*2 | Lob vs N&S |
| C3: Inflammation | s3 | PLI vs. PN&S | s*3 | Lob vs N&S |
| C4: Fibrosis | s4 | PLI vs. PN&S | s*4 | Lob vs N&S |
| C1: Insulin resistance and oxidative stress | s5 | PF vs. PN&S | s*5 | Fib vs N&S |
| C2: cell stress, apoptosis and lipotoxicity | s6 | PF vs. PN&S | s*6 | Fib vs N&S |
| C3: Inflammation | s7 | PF vs. PN&S | s*7 | Fib vs N&S |
| C4: Fibrosis | s8 | PF vs. PN&S | s*8 | Fib vs N&S |
| C1: Insulin resistance and oxidative stress | s9 | PF vs. PLI | s*9 | Fib vs Lob |
| C2: cell stress, apoptosis and lipotoxicity | s10 | PF vs. PLI | s*10 | Fib vs Lob |
| C3: Inflammation | s11 | PF vs. PLI | s*11 | Fib vs Lob |
| C4: Fibrosis | s12 | PF vs. PLI | s*12 | Fib vs Lob |

**Table S4. Gene signature index (created using Data file S3).**

The 24 gene signatures (**Data file S3)** are composed of 2 sets of 12 signatures, with one set derived from the cluster groupings and the other from the clinical classifications (*). Each set is a unique combination of their respective 3 pairwise comparisons and 4 NAFLD pathway categories (see **Methods** for details on the methodology, see **Figure 3 & S1-S2; Table S3;** and **Data file S1** for the distribution and details of these pathways in the pairwise comparisons) .

| Drug name (DrugBank ID) | Gene signature-query frequency | Unique instances | Gene signature indices (see Table S4) and their disease categorization | Canonical targets |
|---|---|---|---|---|
| **vorinostat (DB02546)** | **5** | **4** | **s11: Inflammation<br>s8: Fibrosis<br>s6: Cell Stress, Apoptosis and Lipotoxicity<br>s3: Inflammation<br>s7: Inflammation** | **HDAC1, HDAC2, HDAC3, HDAC6, HDAC8** |
| **SN-38 (DB05482)** | **5** | **3** | **s7: Inflammation<br>s6: Cell Stress, Apoptosis and Lipotoxicity<br>s2: Cell Stress, Apoptosis and Lipotoxicity<br>s4: Fibrosis<br>s5: Insulin Resistance and Oxidative Stress** | **TOP1** |
| **auranofin (DB00995)** | **5** | **2** | **s3: Inflammation<br>s5: Insulin Resistance and Oxidative Stress<br>s7: Inflammation<br>s4: Fibrosis<br>s8: Fibrosis** | **PRDX5, IKBKB** |
| **PX-12 (DB05448)** | **5** | **2** | **s5: Insulin Resistance and Oxidative Stress<br>s6: Cell Stress, Apoptosis and Lipotoxicity<br>s2: Cell Stress, Apoptosis and Lipotoxicity<br>s3: Inflammation<br>s8: Fibrosis** | **TXNRD1** |
| **methylene-blue (DB08167)** | **4** | **3** | **s4: Fibrosis<br>s8: Fibrosis<br>s7: Inflammation<br>s5: Insulin Resistance and Oxidative Stress** | **ACHE** |
| **teniposide (DB00444)** | **4** | **2** | **s2: Cell Stress, Apoptosis and Lipotoxicity<br>s6: Cell Stress, Apoptosis and Lipotoxicity<br>s7: Inflammation<br>s4: Fibrosis** | **TOP2A** |
| **trichostatin-a (DB04297)** | **3** | **3** | **s3: Inflammation<br>s5: Insulin Resistance and Oxidative Stress<br>s7: Inflammation** | **HDAC8, HDAC7** |
| **camptothecin (DB04690)** | **3** | **2** | **s2: Cell Stress, Apoptosis and Lipotoxicity<br>s6: Cell Stress, Apoptosis and Lipotoxicity<br>s7: Inflammation** | **TOP1** |
| **dexamethasone (DB01234)** | **3** | **2** | **s1: Insulin Resistance and Oxidative Stress<br>s5: Insulin Resistance and Oxidative Stress<br>s4: Fibrosis** | **NR3C1, NR0B1, ANXA1, NOS2, NR1I2** |
| **geldanamycin (DB02424)** | **3** | **2** | **s7: Inflammation<br>s11: Inflammation<br>s4: Fibrosis** | **HSP90AB1, HSP90AA1, HSP90B1** |
| **capsaicin (DB06774)** | **3** | **1** | **s6: Cell Stress, Apoptosis and Lipotoxicity<br>s7: Inflammation<br>s3: Inflammation** | **TRPV1, PHB2** |

| Drug name (DrugBank ID) | Gene signature-query frequency | Unique instances | Gene signature indices (see Table S4) and their disease categorization | Canonical targets |
|---|---|---|---|---|
| **curcumin (DB11672)** | **3** | **1** | **s8: Fibrosis<br>s4: Fibrosis<br>s6: Cell Stress, Apoptosis and Lipotoxicity** | **PPARG, VDR, ABCC5, CBR1, GSTP1** |
| **itraconazole (DB01167)** | **3** | **1** | **s2: Cell Stress, Apoptosis and Lipotoxicity<br>s1: Insulin Resistance and Oxidative Stress<br>s6: Cell Stress, Apoptosis and Lipotoxicity** | **CYP51A1** |
| **midazolam (DB00683)** | **3** | **1** | **s1: Insulin Resistance and Oxidative Stress<br>s6: Cell Stress, Apoptosis and Lipotoxicity<br>s2: Cell Stress, Apoptosis and Lipotoxicity** | **GABRA1, GABRA2, GABRA5, GABRA3, GABRA4, GABRA6** |
| **olaparib (DB09074)** | **3** | **1** | **s8: Fibrosis<br>s4: Fibrosis<br>s7: Inflammation** | **PARP1, PARP2, PARP3** |
| **chlorpromazine (DB00477)** | **2** | **2** | **s4: Fibrosis<br>s3: Inflammation** | **DRD2, DRD1, HTR1A, HTR2A, ADRA1A, ADRA1B, HRH1, KCNH2, DRD3, DRD4, DRD5, HTR2C, ADRA2A, CHRM1, CHRM3, SMPD1, CALM1, ORM1, HTR6, HTR7, HRH4** |
| **fulvestrant (DB00947)** | **2** | **2** | **s1: Insulin Resistance and Oxidative Stress<br>s4: Fibrosis** | **ESR1** |
| **gemcitabine (DB00441)** | **2** | **2** | **s4: Fibrosis<br>s7: Inflammation** | **RRM1, TYMS, CMPK1** |
| **alvocidib (DB03496)** | **2** | **1** | **s1: Insulin Resistance and Oxidative Stress<br>s5: Insulin Resistance and Oxidative Stress** | **CDK2, CDK5, CDK9, CDK1, CDK6, EGFR, CDK4, CDK8, CDK7, PYGM, PYGB, PYGL** |
| **brompheniramine (DB00835)** | **2** | **1** | **s1: Insulin Resistance and Oxidative Stress<br>s6: Cell Stress, Apoptosis and Lipotoxicity** | **HRH1, CHRM1, CHRM2, CHRM3, CHRM4, CHRM5** |
| **cladribine (DB00242)** | **2** | **1** | **s4: Fibrosis<br>s3: Inflammation** | **RRM1, RRM2, RRM2B, POLA1, POLE, POLE2, POLE3, POLE4, PNP** |
| **dasatinib (DB01254)** | **2** | **1** | **s8: Fibrosis<br>s4: Fibrosis** | **ABL1, SRC, EPHA2, LCK, YES1, KIT, PDGFRB, STAT5B, ABL2, FYN, BTK, NR4A3, BCR, CSK, EPHA5, EPHB4, FGR, FRK, HSPA8, LYN, ZAK, MAPK14, PPAT** |
| **dinoprost (DB12789)** | **2** | **1** | **s6: Cell Stress, Apoptosis and Lipotoxicity<br>s5: Insulin Resistance and Oxidative Stress** | **PTGDR2** |

| Drug name (DrugBank ID) | Gene signature-query frequency | Unique instances | Gene signature indices (see Table S4) and their disease categorization | Canonical targets |
|---|---|---|---|---|
| **fexaramine (DB02545)** | **2** | **1** | **s2: Cell Stress, Apoptosis and Lipotoxicity**<br>**s1: Insulin Resistance and Oxidative Stress** | **NR1H4** |
| **fexofenadine (DB00950)** | **2** | **1** | **s5: Insulin Resistance and Oxidative Stress**<br>**s3: Inflammation** | **HRH1** |

**Table S5. 25 highest ranked predicted drugs based on initial CMap analysis.**

For each gene signature (indexed in **Table S4** & **Data file S3, as** signatures: s1-s12), the 20 highest ranking compounds were selected (FDR *p*-value < .05) using their respective most negative CMap score among the perturbation instances from the 2017 LINCS database (*5*) (see **Methods**). Drugs/small molecules perturbagens identified in more than 1 gene signature-based query were prioritized based both on the number of occurrences across the 12 queries and termed: Gene signature-query frequency (**Data Files S4-S5**) and the number of unique LINCS perturbation instances across the gene signatures. Each signature-based query is  ordered (from highest to lowest) according to the relative rank of the drug within each query from which the drug was identified (i.e., occurrence). Each gene signature-based query is associated with a predominate feature (i.e., disease category) of NAFLD. The canonical targets derive from DrugBank (v5.1.4).

| Compound | Target | LogP value (PubChem) | % drug recovery at 72 h | $TC_{50}$ (μM) |
|---|---|---|---|---|
| Obeticholic Acid (OCA) | FXR | 5.1 | 90% | ND |
| Pioglitazone (PGZ) | PPARγ | 3.9 | 95% | ND |
| Vorinostat (SAHA) | HDAC | 1.4 | 86% | 29.8 |

**Table S6. Drug binding and cytotoxicity profiles for compounds used in LAMPS studies.**

To assess the drug binding capability of the polydimethylsiloxane (PDMS)-containing LAMPS device for compounds used in these studies, we used perfusion flow tests and mass spectrometry analysis of efflux collected from LAMPS devices at 72 h to determine the overall effective concentration of each compound compared to the starting concentration of drug as previously described (*12, 14*). The $TC_{50}$ (Toxic Concentration inducing 50% hepatocyte death) was determined in a 5-day hepatocyte cytotoxicity assay (**Expanded Methods**). ND- not determined. The $TC_{50}$ assay was not conducted on Obetacholic acid or Pioglitazone. The concentration of these compounds was based on previous experimentation in the LAMPS model.

| Rank | Gene name | Entrez gene ID | NAFLD Subnetwork Degree | Liver interactome Degree |
|---|---|---|---|---|
| 1 | HSP90AA1 | 3320 | 64 | 354 |
| 2 | FBXO6 | 26270 | 40 | 220 |
| 3 | MAPK1 | 5594 | 35 | 192 |
| 4 | CDK2 | 1017 | 32 | 342 |
| 5 | HSP90AB1 | 3326 | 32 | 213 |
| 6 | IKBKG | 8517 | 31 | 189 |
| 7 | TNFRSF1A | 7132 | 30 | 126 |
| 8 | PIK3R1 | 5295 | 30 | 137 |
| 9 | STAT3 | 6774 | 30 | 134 |
| 10 | MAP3K7 | 6885 | 29 | 122 |
| 11 | HSPA5 | 3309 | 28 | 214 |
| 12 | MAPK8 | 5599 | 27 | 131 |
| 13 | SHC1 | 6464 | 27 | 123 |
| 14 | ATF2 | 1386 | 27 | 118 |
| 15 | MAPK14 | 1432 | 26 | 154 |
| 16 | CASP8 | 841 | 26 | 111 |
| 17 | PRKCZ | 5590 | 25 | 105 |
| 18 | PRKCA | 5578 | 25 | 166 |
| 19 | YWHAE | 7531 | 24 | 162 |
| 20 | STUB1 | 10273 | 24 | 144 |

**Table S7. The 20 highest ranked hubs (proteins/targets) by degree in the NAFLD subnetwork.**

The hubs are indicated by gene name and the degree is defined by the number of interactions with proteins encoded by other NAFLD DEGs. For comparison, the degree of the hub is also indicated in the context of the background human liver protein-protein interactome. This Table was generated using **Data file S6** and provides additional detail to **Figure S5.**

| Rank | Drug name | Z-score | Targets |
|---|---|---|---|
| 1 | isoprenaline (DB01064) | -2.78 | ADRB1, ADRB2, ADRB3, MAPK1, PIK3R1, PIK3R2, PIK3R3, PDE4A, SOD1 |
| 2 | fenoprofen (DB00573) | -2.61 | PTGS2, PTGS1, PPARA, PPARG |
| 3 | streptozotocin (DB00428) | -2.47 | SLC2A2, MGEA5 |
| 4 | palbociclib (DB09073) | -2.27 | CDK4, CDK6 |
| 5 | 7-hydroxystaurosporine (DB01933) | -2.23 | PDPK1 |
| 6 | alvespimycin (DB12442) | -1.96 | HSP90AA1 |
| 7 | k-252a (DB02152) | -1.44 | MET, RNMT, MAP2K1 |
| 8 | adenosine-phosphate (DB00131) | -1.25 | CREB1, PIM1, PDE4B, PYGL, PRKAB1, HINT1, PDE4D, ACSS1, ACSS2, PRKAA1, PRKAB2, ADCY1, ACSL1, FBP1, ADK |
| 9 | alfacalcidol (DB01436) | -1.22 | CYP27B1, VDR, RXRA |
| 10 | cinnarizine (DB00568) | -0.77 | DHX8, HRH1, DHX34, CACNA1C, CACNA1D, CACNA1F, CACNA1S, CACNA1G, CACNA1H, CACNA1I, ENTHD1, DRD2, DRD1, CHRM1 |
| 11 | ambrisentan (DB06403) | -0.75 | EDNRA, EDNRB |
| 12 | hexestrol (DB07931) | -0.61 | AKR1C1, ESR1, NR1I2, NR1I3 |
| 13 | nifedipine (DB01115) | -0.55 | CACNA1C, CACNA2D1, CACNB2, CACNA1D, CACNA1S, CALM1, KCNA1, CACNA1H, NR1I2 |
| 14 | mifepristone (DB00834) | -0.31 | PGR, NR3C1, KLK3, KLKB1, NR1I2 |
| 15 | fluvastatin (DB01095) | -0.26 | HMGCR |
| 16 | mevastatin (DB06693) | -0.26 | HMGCR |
| 17 | cytarabine (DB00987) | -0.19 | POLB, POLG2 |
| 18 | ephedrine (DB01364) | 0.12 | SLC6A2, ADRA1D, ADRA1A, SLC18A2, ACHE |
| 19 | ethinylestradiol (DB00977) | 0.14 | ESR1, NR1I2, SHBG |

| Rank | Drug name | Z-score | Targets |
|---|---|---|---|
| **20** | **tetracycline (DB00759)** | **0.14** | **PRNP, PADI4** |
| **21** | **fluocinolone (DB00591)** | **0.21** | **NR3C1, ANXA1, ANXA2, ANXA3, ANXA4, ANXA5** |
| **22** | **indirubin (DB12379)** | **0.24** | **CYP1A1, AHR** |
| **23** | **dopamine (DB00988)** | **0.27** | **DRD2, DRD1, DRD5, DRD3, DRD4, SLC6A3, DBH, HTR1A, HTR7, SLC6A2, SLC6A4, HTR3A, HTR3B, SOD1, SLC18A2** |
| **24** | **flucytosine (DB01099)** | **0.28** | **DNMT1** |
| **25** | **vemurafenib (DB08881)** | **0.34** | **BRAF** |

**Table S8. Prioritization of CMap-predicted drugs and small-molecule perturbagens based on NAFLD subnetwork proximity.**

This table is derived from **Data file S7.** The common name of the drug/small molecule with the DrugBank ID in parenthesis is shown. The Z-scores were calculated as described in the **Methods** and Guney et al.(*18*). The targets are extracted from DrugBank (v5.1.4), those in red are directly in the NAFLD subnetwork (**Figure S6**; **Table S7**, and **Data file S6**). The CMap analysis was performed precisely as described in Table 1.

**Data file S1. Differentially enriched pathways for each pairwise cluster and clinical classification comparison (**Link to csv file**).**

These results were used to create **Table S3**, the gene signatures (**Table S4; Data file S3**)**.** See **Methods** for details**.** The columns of this file are as follows:

- comparison: The pairwise comparison
- pathway_name:
- id: KEGG pathway ID
- KEGG pathway group
- KEGG pathway subgroup
- nafld_categories: Denotes the involvement of the pathway in NAFLD pathophysiology (see **Methods**).
    - C1: Insulin resistance and oxidative stress
    - C2: cell stress, apoptosis and lipotoxicity
    - C3: Inflammation
    - C4: Fibrosis
    - C5: Disease related pathways
    - C6: Other associated pathways
    - C7: No established relationship
- logFC: estimate of the log2-fold-change of the comparison (see limma documentation)
- CI.L: LogFC 95% confidence interval lower limit (see limma documentation)
- CI.R: LogFC 95% confidence interval upper limit  (see limma documentation)
- AveExpr: average log2-expression across all (see limma documentation)
- t: moderated t-statistic (see limma documentation and Smyth (*19*))
- P.Value: raw *p*-value (see limma documentation)
- adj.P.Val: FDR corrected *p*-value (see limma documentation)
- B: log-odds that the gene is differentially expressed (see limma documentation)
- pmids: The PMIDs for the references which support the NAFLD categorization

**Data file S2. DEGs resulting for each pairwise cluster and clinical classification comparisons (Link to csv file).**

These results were used in the creation of gene signatures (**Table S4; Data file S3**) and NAFLD subnetwork ( **Figure S5**, **Table S7; Data file S6**). The columns of this file are as follows:

- comparison: The pairwise comparison
- gene_symbol: Common gene name
- Entrez gene ID
- Ensembl gene ID
- logFC: estimate of the log2-fold-change of the comparison (see limma documentation)
- CI.L: LogFC 95% confidence interval lower limit (see limma documentation)
- CI.R: LogFC 95% confidence interval upper limit (see limma documentation)
- AveExpr: average log2-expression across all samples (see limma documentation)
- t: moderated t-statistic (see limma documentation and Smyth (*19*))
- P.Value: raw *p*-value (see limma documentation)
- adj.P.Val: FDR corrected p-value (see limma documentation)
- B: log-odds that the gene is differentially expressed (see limma documentation)
- kegg_pathway_names: The names of the KEGG pathways that the gene is a member of (if applicable, NA otherwise)
- kegg_pathway_ids: The pathway ids the KEGG pathways that the gene is a member of (if applicable, NA otherwise)

**Data file S3. Gene signatures used for CMap analysis (Link to csv file).**

The data from **Data files S1-S2** were used to create this file (see **Methods**). It was used for CMap drug prediction (**Tables 1 & S5; Data file S4-S5**, see **Methods** for details on the methodology). The columns are as follows:

- gene_sig_idx: The gene signature index (see **Table S4** and **Data file S3**)
- comparison: The pairwise comparison
- nafld_pathway_category: The NAFLD category of differentially enriched pathways that was used to create the gene signature (see **Methods**), The values are defined as follows:
  - C1: Insulin resistance and oxidative stress
  - C2: cell stress, apoptosis and lipotoxicity
  - C3: Inflammation
  - C4: Fibrosis
- up-regulated_gene_names: List of the upregulated genes (using common gene name) for the signature
- up-regulated_entrez_ids: List of the upregulated genes (using entrez gene id) for the signature
- down-regulated_gene_names: List of the down-regulated genes (using common gene name) for the signature
- down-regulated_entrez_ids: List of the down-regulated genes (using entrez gene id) for the signature

**Data file S4. CMAP scores of small molecules with a DrugBank ID for the 24 queries described in the Methods (Link to csv file).**

These results were used to create **Table 1 & S5** (see **Methods** for details ). columns are as follows:

- gene_sig_idx: The gene signature index (see **Table S4** and **Data file S3**)
- comparison: The pairwise comparison
- nafld_pathway_category: The NAFLD category of differentially enriched pathways that was used to create the gene signature (see **Methods**), The values are defined as follows:
    - C1: Insulin resistance and oxidative stress
    - C2: cell stress, apoptosis and lipotoxicity
    - C3: Inflammation
    - C4: Fibrosis
- sig_id: The L100 perturbation instance signature id (see the GEO CMap LINCS user guide for more information)
- lincs_db: The database (2017, 2020, or both) from which the perturbation instance originates
- pert_id: The Broad's internal drug/small molecule ID (see the GEO CMap LINCS user guide for more information)
- pert_iname: The Broad's drug/small molecule common name (see the GEO CMap LINCS user guide for more information)
- drugbank_id: DrugBrank's drug/small molecule ID
- targets: The drug/small molecule targets from DrugBank v5.1.4
- cmap_score: The CMap score (see **Methods** and (*5, 20*))
- p_value: *P*-value calculated by permutation testing (see Chen et al(*21*))
- fdr_*p*-value: False discovery rate corrected *p*-value

**Data file S5. List of top 20 CMap predictions from both the 2017 & 2020 LINCS databases and both ranking methods ("Best score" and "Percentile score") from the 24 signatures (link to file).**

These results were created from **Data file S4** and were used to create **Table 1** and **Table S5.** The columns are as follows:

- gene_sig_idx: The gene signature index (see **Table S4** and **Data file S3**)
- comparison: The pairwise comparison
- nafld_pathway_category: The NAFLD category of differentially enriched pathways that was used to create the gene signature (see **Methods**), The values are defined as follows:
    - C1: Insulin resistance and oxidative stress
    - C2: cell stress, apoptosis and lipotoxicity
    - C3: Inflammation
    - C4: Fibrosis
- lincs_db: The database (2017 either 2020) from which the perturbation instance originates
- pert_id: The Broad's internal drug/small molecule ID (see the GEO CMap LINCS user guide for more information)
- pert_iname: The Broad's drug/small molecule common name (see the GEO CMap LINCS user guide for more information)
- drugbank_id: DrugBrank's drug/small molecule ID
- summary_stat: Which compound-centric statistic used to rank the compounds ( either best_score or prct_67th_score, see **Methods**)
- drug_rank: Relative rank of the compound prediction within the gene signature
- pert_sum_score: The summary score used to rank the compound (see **Methods**)

**Data file S6. Degree of the nodes in the NAFLD subnetwork (Figure S5, Link to csv file).**

These results are discussed in the **Results** section of the main text and supports **Table S7.** The columns are as follows:

- gene_symbol: The common gene name
- gene_description
- Entrez_gene_id
- degree_liver: The number of connections this protein has to other nodes in the human liver interactome
- degree_nafld_DEGs: The number of connections the encoded protein has with other DEG encoded nodes in the NAFLD associated network

**Data file S7. Network proximity determined Z-scores for the highest ranking CMap-predicted drugs with targets mapping to the NAFLD subnetwork (Link to csv file).**

These results were used for **Table S8**. The columns are as follows:

- drug_name: Common name of the drug/small molecule
- drugbank_id: DrugBank ID of the drug/small molecule
- z: Z-score of the normalized distance of drug subnetwork to disease associated subnetwork (See **Methods**, (*18*))
- d: Shortest distance of drug subnetwork to disease associated subnetwork (See **Methods**, (*18*))
- mean: Average distance of a reference network to disease associated subnetwork (See **Methods**, (*18*))
- sd: Standard deviation of a reference network to disease associated subnetwork (See **Methods**, (*18*))

**Data file S8. DEGs resulting from the LAMPS pairwise comparisons (EMS vs NF, LMS vs NF, LMS vs EMS) (Link to csv file).**

These results were used to create **Data file S9.** The columns of this file are as follows:

- comparison: The pairwise comparison
- gene_symbol: Common gene name
- Entrez gene ID
- Ensembl gene ID
- logFC: estimate of the log2-fold-change of the comparison (see limma documentation)
- CI.L: LogFC 95% confidence interval lower limit (see limma documentation)
- CI.R: LogFC 95% confidence interval upper limit (see limma documentation)
- AveExpr: average log2-expression across all samples (see limma documentation)
- t: moderated *t*-statistic (see limma documentation and Smyth (*19*))
- P.Value: raw *p*-value (see limma documentation)
- adj.P.Val: FDR corrected *p*-value (see limma documentation)
- B: log-odds that the gene is differentially expressed (see limma documentation)
- kegg_pathway_names: The names of the KEGG pathways that the gene is a member of (if applicable, NA otherwise)
- kegg_pathway_ids: The pathway ids the KEGG pathways that the gene is a member of (if applicable, NA otherwise)

**Data file S9. Differentially enriched pathways the LAMPS pairwise comparisons (EMS vs NF, LMS vs NF, LMS vs EMS) (Link to csv file).**

These results along with **Data file S1** were used for **Figure S5.** See **Methods** for details**.** The columns of this file are as follows:

- comparison: The pairwise comparison
- pathway_name:
- id: KEGG pathway ID
- KEGG pathway group
- KEGG pathway subgroup
- nafld_categories: Denotes the involvement of the pathway in NAFLD pathophysiology (see **Methods**).
    - C1: Insulin resistance and oxidative stress
    - C2: cell stress, apoptosis and lipotoxicity
    - C3: Inflammation
    - C4: Fibrosis
    - C5: Disease related pathways
    - C6: Other associated pathways
    - C7: No established relationship
- NES: normalized enrichment score (see clusterProfiler documentation)
- pvalue: uncorrected p-value from permutation testing (see clusterProfiler documentation)
- p.adjust: FDR corrected *p*-values (see clusterProfiler documentation)

**Data file S10. The 71 features selected by the final MLENet model (Link to csv file).**

These results were used to supplement **Figure 4.** See **Methods** for details**.** The columns of this file are as follows:

- class: the patient clinical classification (Normal, Steatosis, Lobular inflammation, or Fibrosis)
- ensembl_gene_id
- gene_name
- Entrez gene ID
- estimate: the feature coefficients (i.e., association with each class) estimated by MLENet (see glmnet documentation)
- prior_nafld_association: The PMIDs for papers references associating the feature with NAFLD or comparative toxicogeneomics database disease association

**Expanded Methods**

**Cell sources and culture**

A single lot of selected cryopreserved primary human hepatocytes (lot# HU1960) with >90% viability and re-plating efficiency post-thaw were purchased from ThermoFisher. Human liver sinusoidal endothelial cells (LSECs) were purchased from LifeNet Health. The human monoblast cell line, THP-1, used to generate Kupffer cells, was purchased from ATCC and LX-2 human stellate cells were purchased from EMD Millipore. LSECs were cultured in endothelial cell basal medium-2 (EBM-2) supplemented with the endothelial growth medium-2 (EGM-2) supplement pack (Lonza). THP-1 cells were cultured in suspension in RPMI-1640 medium (ThermoFisher) supplemented with 10% fetal bovine serum (FBS; ThermoFisher), 100 µg/mL penicillin streptomycin (ThermoFisher), and 2 mM L-glutamine (ThermoFisher). THP-1 cells were differentiated into mature macrophages by treatment with 200 ηg/mL phorbol myristate acetate (Sigma Aldrich) for 48 h. LX-2 cells were cultured in Dulbecco's Modified Eagle Medium (DMEM; ThermoFisher) supplemented with 2% FBS and 100 µg/mL penicillin streptomycin.

**Normal fasting and Early Metabolic Syndrome (EMS) media**.

We recently developed MPS culture media conditions to create disease progression from Normal Fasting (NF) to early metabolic syndrome (EMS) over a two-week period in the LAMPS platform (*17*) that recapitulates key features of the NAFLD disease process. We developed the media around Williams E media that did not have glucose, insulin, glucagon, oleic acid, palmitic acid and then adjusted these components to reflect the pathophysiological conditions.

*Normal Fasting (NF) Media:* NF media was prepared in a custom formulation of William's E medium without glucose (ThermoFisher) supplemented with 5.5 mM glucose (Sigma Millipore), 1% FBS (Corning), 0.125 g/mL bovine serum albumin (Sigma), 0.625 mg/mL human transferrin, 0.625 µg/mL selenous acid, 0.535 mg/mL linoleic acid (Sigma), 100 nM dexamethasone (ThermoFisher), 2 mM glutamax, 15 mM HEPES (ThermoFisher), 100 U/100 µg/mL pen/ strep (Hyclone Labs), 10 pM insulin (ThermoFisher) and 100 pM glucagon (Sigma).

*EMS Media:* Early metabolic syndrome (EMS) medium was derived from the NF media formulation with the following modifications: 11.5 mM glucose, 10 nM insulin, 30 pM glucagon, 200 µM sodium oleate (Sigma) and 100 µM palmitate (Cayman Chemical Company).

**LAMPS model assembly and maintenance workflow.**

**Day -3:**

(a) *Mixed matrix coating of MPS devices:* The interior of the devices was dried under vacuum prior to protein coating with 100 µg/mL bovine fibronectin (Sigma Millipore) and 150 µg/mL rat-tail collagen, type 1(Corning), in PBS for 1 h at room temperature. The collagen/fibronectin solution was then removed, and devices were filled with PBS and stored at 4°C until use.
(b) *Differentiation of THP-1 cells:* THP-1 cells were treated with 200 ηg/mL phorbol myristate acetate (PMA; Sigma Millipore) to facilitate their differentiation into mature macrophages for seeding into LAMPS models on Day -1 (48 h treatment).

**Day -2:**

(a) *Hepatocyte seeding:* Cryopreserved hepatocytes were thawed following the manufacturer's recommendations. Hepatocytes were pelleted at 100 x g for 10 minutes using Cryopreserved Hepatocyte Recovery Medium (CHRM; ThermoFisher), and then resuspended at 2.75 x $10^6$ hepatocytes/mL in hepatocyte plating media (HPM). Hepatocyte cell solution was then injected into the interstitial compartment of the device for overnight incubation at 37°C to allow for cell adherence and spreading.

**Day -1:**

(a) *LECM coating of MPS devices:* HPM was removed from the device and a solution of 400 µg/ml of porcine liver extracellular matrix prepared in NF media (LECM; a kind gift from Dr. Stephen Badylak's laboratory at the McGowan Institute for Regenerative Medicine, University of Pittsburgh) was added and incubated for 3 h at 37°C to create a thin matrix layer on top of the hepatocytes to mimic the Space of Disse.

(b) *LSEC and THP-1 seeding:* During the LECM incubation, LSEC and THP-1 cell suspensions are prepared in NF media for seeding into LAMPS. LSECs were thawed and a cell suspension was prepared at a concentration of 3.0 x $10^6$ cells/mL. Differentiated THP-1 cells were prepared at a concentration of 1.6 x $10^6$ cells/mL. The individual cell solutions were combined at a 1:1 ratio to yield final cell concentrations of 1.5 x $10^6$ (LSEC) and 0.8 x $10^6$ (THP-1) cells/mL. LECM solution was removed by gentle aspiration using a 1 mL syringe with a blunt needle (Fisher Scientific) and the LSEC/THP-1 cell solution was injected into each device and incubated for 2 h at 37°C.

(c) *Collagen/LX-2 overlay:* LX-2 cells were prepared at a concentration of 0.2 x $10^6$ cells/mL and were suspended in 1 mL of a 2.5 mg/mL solution of pH 7.2 collagen I/10 mM HEPES/HBSS and injected into devices. The devices were then inverted for 1 h at 37°C during collagen polymerization to ensure an initial spatial separation of hepatocytes and LX-2 stellate cells. The devices were then re-inverted and incubated overnight at 37°C. The collagen overlay functions to maintain hepatocyte morphology and functionality over extended culture time.

***Day 0:***

(a) *Establishment of flow:* The next day, flow was initiated using pressure driven pumps (KD Scientific) to perfuse media in glass syringes (Hamilton) at a flow rate of 5 (3-6% $O_2$) µL/hour to achieve a target oxygen concentration of 3-6% $O_2$, corresponding to zone 3 (hepatic venule) oxygen levels, as previously described (*13*). Devices were then maintained for 10 days at this flow rate.

(b) *Drug testing in LAMPS.* For drug studies, EMS media was prepared as described above and supplemented with the indicated concentration of drug (0.1% DMSO v/v final concentration). EMS media containing drug was added at Day 0 during the initiation of flow for the duration of the experimental time course. . The following drug treatments were used in these studies: 10 µM obeticholic acid (Selleck Chemicals), 30 µM pioglitazone (Selleck Chemicals), and 1.7 µM or 5 µM vorinostat (Selleck Chemicals). For drug combination studies, 30 µM pioglitrazone was combined with either 1.7 µM or 5 µM vorinostat for the duration of the experimental time course.

**Drug binding/recovery in PDMS-containing LAMPS device.** To assess the drug binding capability of the polydimethylsiloxane (PDMS)-containing LAMPS device for compounds used in these studies, we used perfusion flow tests and mass spectrometry analysis of efflux collected from LAMPS devices at 72 h to determine the overall effective concentration of each compound compared to the starting concentration of drug as previously described (*12, 14*). Briefly, Nortis devices were coated for 1h at RT with 150 µg/mL collagen I and 100 µg/mL fibronectin solution in PBS. Following this, the collagen/fibronection solution was removed and devices were washed 2x with sterile PBS. A 2.5 mg/mL collagen I overlay solution was prepared in perfusion media and injected into each device where they were incubated at 37°C o/n. The next day, drug solutions were prepared at the desired concentrations in EMS media for each compound and loaded into 10 mL glass syringes. A flow rate of 15 µl/h was established, and efflux media was collected at 24, 48, and 72 h of flow and the amount of compound at the 72 h time point was compared to the amount of drug present from the starting solution. Mass spectrometry was then performed by the University of Pittsburgh Small Molecule Biomarker Core where data was collected with a Waters Acquity UPLC (Milford, MA) C18, 1.7 µm, 2.1 X 100 mm reversed-phase column. Separation was carried out in an acetonitrile: water (0.1% formic acid) gradient and detection and quantitation were achieved in the positive ion mode with a TSQ Quantum Ultra Mass Spectrometer interfaced via an electrospray ionization (ESI) probe. Recovery was calculated as the ratio of Efflux Area Under Curve/Influx Area Under Curve.

**5 Day Cytotoxicity Assay.** Primary human hepatocytes (Thermo Fisher lot Hu1981) were thawed and resuspended in Hepatocyte Recovery Media (Thermo Fisher CM4000). Viable hepatocytes were collected by centrifugation (100g X 11 minutes). The supernate was removed by aspiration and the hepatocyte pellet resuspended in Hepatocyte Plating Media, counted and then 14,000 hepatocytes/well (560,000 hepatocytes/ml) were seeded at into a collagen 1 pre-coated 384 well microtiter plate (Becton Dickinson). The cells were allowed overnight attachment and spreading. The plate was decanted before 25 µl of 1.5% gelling collagen 1/NF media solution added to each well. The gel polymerized 1 hour (37$^{o}$ C, 5% $CO_2$) before the addition of 25 µl microtiter plate with NF media ± 2X concentration of compound. The plate was incubated 5 days with a single replenishment of 25 µl NF media ± 1X concentration of compound added to each well at 48 hr. Cytotoxicity was assessed by propidium iodide uptake assay. A 4X solution of propidium iodide (PI) and Hoechst nuclear dye was prepared in NF media at 8 µg/ml Hoechst and 20 µg/ml PI. 25 µl of the PI/Hoechst solution was added to each well for 1 hour. Fluorescent images were collected for Hoechst (405/488 ex/em) and PI (488/530 ex/em) using a High Content Screening (HCS) instrument. The compartment analysis algorithm software of the HCS was used to quantitate the intensity of PI co-localized within the nucleus and calculate the % of PI positive hepatocytes.

**Secretome measurements.** Efflux media from LAMPS devices was collected on days 2,4,6,8, and 10 to measure albumin, blood urea nitrogen, and lactate dehydrogenase. The enzyme linked immunosorbent assay (ELISA) for albumin was purchased from Bethyl Laboratories. The CytoTox 96 for lactate dehydrogenase (LDH) and the urea nitrogen test were purchased from Promega and Stanbio Laboratory, respectively. Collagen 1A1 (R&D Systems and TIMP-1 ELISA measurements were made from day 10 efflux only. All efflux measurements were obtained as described previously (*12-14, 22*).

**Steatosis measurements**. Steatosis measurements were performed after completion of the experimental time course (Day 10) in LAMPS models as previously described (*13, 17*). Cells were fixed with 4% paraformaldehyde in PBS for 30 min then washed twice with PBS for 10 min. Following fixation, HCS LipidTOX Deep Red Neutral Lipid Stain (ThermoFisher) was diluted 1:500 in PBS and perfused into devices and incubated overnight at 4°C. The following day, devices were washed twice with PBS and then incubated for 15 min with 5 µg/mL Hoechst (ThermoFisher) to label nuclei. Images were collected with a Nikon 20x (0.45 NA) objective using the IN Cell Analyzer 6000 (GE Healthcare) in confocal mode using the 405 nm (Hoechst) and 640 nm (LipidTOX) lasers and associated filter sets with the aperture set to 1 airy unit and were acquired with a sCMOS 5.5 Mp camera (2560 x 2160 pixels). Images for each media treatment were acquired using the same exposure time (100 ms) and laser power (80%) settings to ensure that intensity values were ~50-75% of the total dynamic range of the InCell system (65,000 bits). Additionally, imaging parameters were set using the EMS media vehicle control because this condition served as the positive control, demonstrating the most LipidTOX staining. Z-stacks totaling 100 µm distance (5 µm spacing between slices) were obtained and then imported into FIJI (ImageJ) to generate maximum intensity projections. Lipid droplets were identified using FIJI by interactive selection of a threshold (default method) using uniform intensity minimum (background) and intensity maximum values across the image sets. Watershed segmentation and the analyze particles function was then used to measure the total lipid intensity in each device. Intensity values were then normalized on a per cell basis by counting the total number of Hoechst-positive nuclei per field using the cell counting function in FIJI. A total of 10 images per device were collected from n =3 devices for each experimental condition. Statistical significance of LipidTOX labeling was assessed using a One-Way ANOVA with Tukey's multiple comparisons test to make comparisons between each control and drug treatment group, where *p*-values less than 0.05 were considered statistically significant.

**Stellate cell activation.** Staining for LX-2 cell expression of α-smooth muscle actin (α-SMA) was performed after completion of the experimental time course (Day 10) in LAMPS models as previously described (*17*). Cells were fixed with 4% paraformaldehyde in PBS for 30 min, washed twice with PBS for 10 min, then were permeabilized for 30 min. with 0.1% TX-100 in PBS and blocked for an additional 1 h in PBS containing 1% BSA. Mouse monoclonal anti-α-SMA antibody (Sigma Millipore) was diluted 1:100 in PBS containing 0.5% BSA and was incubated overnight at 4°C. The primary antibody was rinsed 3 times for 15 min. with PBS before the cells were then incubated for 1 h with Alexa FluorÒ Goat anti-mouse 555 (ThermoFisher) secondary antibody diluted 1:250 in PBS. The secondary antibody solution was removed, and cells were incubated for 15 min with PBS containing 5 µg/mL Hoechst and then washed 2 more times for 10 minutes with PBS. Images were collected with a Nikon 20x (0.45 NA) objective using the IN Cell Analyzer 6000 (GE Healthcare) in confocal mode using the 405 nm (Hoechst) and 561 nm (α-SMA) lasers and associated filter sets with the aperture set to 1 airy unit. Images for each media treatment were acquired using the same exposure time and laser power settings. Z-stacks totaling 100 µm distance (5 µm spacing between slices) were obtained and then imported into FIJI to generate maximum intensity projections. Image analysis of LX-2 α-SMA expression was quantified using an interactive selection of threshold (Default mode) to mask α-SMA-specific fluorescence. The analyze particles function was then used with a size exclusion

setting of 100 $\mu m^2$ to exclude non-specific staining to measure the integrated intensity of α-SMA expression. A total of 10 images per device were collected from n = 3 devices for each experimental condition. Statistical significance of α-SMA intensity was assessed using a One-Way ANOVA with Tukey's multiple comparisons test test to make comparisons between each control and drug treatment group, where *p*-values less than 0.05 were considered statistically significant.

**Multiplex immunoassays.** Day 10 efflux media from LAMPS devices was collected for each drug treatment group and the levels of various human cytokines (IL-1b, IL-6, IL-8, TNF-a, and MCP-1) were assayed using a custom version (5-plex) of the Human XL Cytokine Discovery Panel (R&D systems). Assays were completed according to the manufacturer's instructions at The University of Pittsburgh Cancer Proteomics Facility Luminex® Core Laboratory. All multiplex panels were run at the same time to avoid run-to-run and operator error variability utilizing the xMAP platform licensed by Luminex®. All the cytokine target profiling experiments were performed from efflux obtained from n = 3 devices for each drug treatment condition. Statistical significance of cytokine secretion between treatment groups was assessed using a One-Way ANOVA with Dunnett's test to make comparisons between each drug treatment group and the vehicle control, where *p* values less than 0.05 were considered statistically significant.

**References**


1. D. R. Zerbino *et al.*, Ensembl 2018. *Nucleic Acids Research* **46**, D754-D761 (2017).
2. G. S. Gerhard *et al.*, Transcriptomic Profiling of Obesity-Related Nonalcoholic Steatohepatitis Reveals a Core Set of Fibrosis-Specific Genes. *Journal of the Endocrine Society* **2**, 710-726 (2018).
3. D. Lefever. (2021).
4. A. Liberzon *et al.*, Molecular signatures database (MSigDB) 3.0. *Bioinformatics* **27**, 1739-1740 (2011).
5. A. Subramanian *et al.*, A Next Generation Connectivity Map: L1000 Platform and the First 1,000,000 Profiles. *Cell* **171**, 1437--1452 (2017).
6. R. S. Marinka Zitnik, Sagar Maheshwari,, J. Leskovec. (2018).
7. D. S. Wishart *et al.*, DrugBank 5.0: a major update to the DrugBank database for 2018. *Nucleic Acids Res* **46**, D1074-d1082 (2018).
8. S. Hanzelmann, R. Castelo, J. Guinney, GSVA: gene set variation analysis for microarray and RNA-seq data. *BMC Bioinformatics* **14**, 7 (2013).
9. M. Ahrens *et al.*, DNA methylation analysis in nonalcoholic fatty liver disease suggests distinct disease-specific and remodeling signatures after bariatric surgery. *Cell Metab* **18**, 296-302 (2013).
10. B. M. Arendt *et al.*, Altered hepatic gene expression in nonalcoholic fatty liver disease is associated with lower hepatic n-3 and n-6 polyunsaturated fatty acids. *Hepatology* **61**, 1565-1578 (2015).
11. S. K. Murphy *et al.*, Relationship between methylome and transcriptome in patients with nonalcoholic fatty liver disease. *Gastroenterology* **145**, 1076--1087 (2013).
12. L. A. Vernetti *et al.*, A human liver microphysiology platform for investigating physiology, drug safety, and disease models. *Experimental biology and medicine (Maywood, N.J.)* **241**, 101-114 (2016).
13. F. T. Lee-Montiel *et al.*, Control of oxygen tension recapitulates zone-specific functions in human liver microphysiology systems. *Experimental biology and medicine (Maywood, N.J.)* **242**, 1617-1632 (2017).
14. M. T. Miedel *et al.*, Modeling the Effect of the Metastatic Microenvironment on Phenotypes Conferred by Estrogen Receptor Mutations Using a Human Liver Microphysiological System. *Scientific reports* **9**, 8341 (2019).
15. C. Sakolish *et al.*, Analysis of reproducibility and robustness of a human microfluidic four-cell liver acinus microphysiology system (LAMPS). *Toxicology* **448**, 152651 (2021).
16. A. Gough *et al.*, Human biomimetic liver microphysiology systems in drug development and precision medicine. *Nat Rev Gastroenterol Hepatol* **18**, 252-268 (2021).
17. M. Saydmohammed *et al.*, Quantifying the progression of non-alcoholic fatty liver disease in human biomimetic liver microphysiology systems with fluorescent protein biosensors. *Experimental biology and medicine (Maywood, N.J.)*, 15353702211009228 (2021).
18. E. Guney, J. Menche, M. Vidal, A.-L. Barábasi, Network-based in silico drug efficacy screening. *Nature Communications* **7**, 10331 EP (2016).
19. G. K. Smyth, Linear models and empirical bayes methods for assessing differential expression in microarray experiments. *Statistical applications in genetics and molecular biology* **3**, Article3 (2004).

20. J. Lamb *et al.*, The Connectivity Map: using gene-expression signatures to connect small molecules, genes, and disease. *Science* **313**, 1929-1935 (2006).
21. B. Chen *et al.*, Computational Discovery of Niclosamide Ethanolamine, a Repurposed Drug Candidate That Reduces Growth of Hepatocellular Carcinoma Cells In Vitro and in Mice by Inhibiting Cell Division Cycle 37 Signaling. *Gastroenterology* **152**, 2022-2036 (2017).
22. X. Li, S. M. George, L. Vernetti, A. H. Gough, D. L. Taylor, A glass-based, continuously zonated and vascularized human liver acinus microphysiological system (vLAMPS) designed for experimental modeling of diseases and ADME/TOX. *Lab on a chip* **18**, 2614-2631 (2018).
